# *ATG5* cancer mutations and alternative mRNA splicing reveal a conjugation switch that regulates ATG12–ATG5-ATG16L1 complex assembly and autophagy

**DOI:** 10.1038/s41421-019-0110-1

**Published:** 2019-08-27

**Authors:** Daric J. Wible, Hsueh-Ping Chao, Dean G. Tang, Shawn B. Bratton

**Affiliations:** 1 Department of Epigenetics and Molecular Carcinogenesis, The University of Texas MD Anderson Cancer Center, Smithville, TX 78957 USA; 2 Department of Pharmacology and Therapeutics, Roswell Park Cancer Institute, Buffalo, NY 14263 USA; 3 Cancer Stem Cell Institute, Research Center for Translational Medicine, East Hospital, Tongji University School of Medicine, Shanghai, 200120 China

**Keywords:** Ubiquitylation, Autophagosomes, Protein quality control, Macroautophagy

## Abstract

Autophagy is critical for maintaining cellular homeostasis during times of stress, and is thought to play important roles in both tumorigenesis and tumor cell survival. Formation of autophagosomes, which mediate delivery of cytoplasmic cargo to lysosomes, requires multiple autophagy-related (ATG) protein complexes, including the ATG12–ATG5-ATG16L1 complex. Herein, we report that a molecular ATG5 “conjugation switch”, comprised of competing ATG12 and ubiquitin conjugation reactions, integrates ATG12–ATG5-ATG16L1 complex assembly with protein quality control of its otherwise highly unstable subunits. This conjugation switch is tightly regulated by ATG16L1, which binds to free ATG5 and mutually protects both proteins from ubiquitin conjugation and proteasomal degradation, thereby instead promoting the irreversible conjugation of ATG12 to ATG5. The resulting ATG12–ATG5 conjugate, in turn, displays enhanced affinity for ATG16L1 and thus fully stabilizes the ATG12–ATG5-ATG16L1 complex. Most importantly, we find in multiple tumor types that ATG5 somatic mutations and alternative mRNA splicing specifically disrupt the ATG16L1-binding pocket in ATG5 and impair the essential ATG5-ATG16L1 interactions that are initially required for ATG12–ATG5 conjugation. Finally, we provide evidence that ATG16L2, which is overexpressed in several cancers relative to ATG16L1, hijacks the conjugation switch by competing with ATG16L1 for binding to ATG5. While ATG16L2 stabilizes ATG5 and enables ATG12–ATG5 conjugation, this endogenous dominant-negative inhibitor simultaneously displaces ATG16L1, resulting in its proteasomal degradation and a block in autophagy. Thus, collectively, our findings provide novel insights into ATG12–ATG5-ATG16L1 complex assembly and reveal multiple mechanisms wherein dysregulation of the ATG5 conjugation switch inhibits autophagy.

## Introduction

Macroautophagy (hereafter referred to as autophagy) is a highly conserved catabolic process that maintains cellular homeostasis by targeting excess or damaged organelles, large protein aggregates, invading pathogens, and nonselective portions of the cytoplasm for lysosomal degradation via double-membrane vesicles, termed autophagosomes. Initially, a cup-shaped precursor membrane, or phagophore, elongates and envelops cytoplasmic cargo before closing to form a mature autophagosome, which in turn fuses with lysosomes whereupon the inner autophagosomal membrane and sequestered material are degraded by acid hydrolases^1^. Formation of autophagosomes requires two ubiquitin-like conjugation reactions involving autophagy-related 12 (ATG12) and members of the microtubule-associated protein 1 light chain 3 (LC3) family (homologs of yeast Atg8). Like ubiquitin, glycine residues at the immediate C-termini of ATG12 and LC3 family proteins, the latter of which are exposed following cleavage by ATG4 cysteine proteases, are first activated by the E1-like enzyme, ATG7, and then transferred to E2-like enzymes, ATG10 and ATG3, respectively^2,3^. The ATG12~ATG10 intermediate, which possesses a high-energy thioester bond, subsequently binds to ATG5 and, independently of any known E3-like enzyme, facilitates the conjugation of ATG12 to ATG5 at Lys-130^4^.

As there are no known deconjugation enzymes, ATG12–ATG5 conjugates are thought to be irreversibly formed and recruited to phagophores by dimeric ATG16L1, in conjunction with its binding partners, WD repeat domain phosphoinositide-interacting protein 2 (WIPI2) and/or RB1 inducible coiled-coil 1 (RB1CCI, also known as FIP200), thereby forming the heteromeric ATG12–ATG5-ATG16L1 complex^3,5–7^. LC3~ATG3 intermediates are recruited to phagophores through a direct interaction with ATG12 and/or through a membrane curvature-sensing domain in ATG3^8–11^. The ATG12–ATG5-ATG16L1 complex subsequently acts as an E3-like enzyme to catalyze the conjugation of LC3 to phosphatidylethanolamine (PE) on the phagophore membrane^12^. PE-conjugated LC3 family proteins recruit diverse cytoplasmic cargoes to phagophores, either directly or through adapter proteins such as p62 (also known as SQSTM1)^13^. They are also implicated in the recruitment of the unc-51 like autophagy activating kinase 1 (ULK1) complex and the expansion and closure of phagophores^14^, as well as the fusion of mature autophagosomes with lysosomes^15^. While the basic ATG12 and LC3 ubiquitin-like conjugation reactions have been characterized, the mechanisms regulating ATG12 conjugation and ATG12–ATG5-ATG16L1 complex assembly remain unclear.

Preclinical data from multiple *Atg* gene knockout mouse models suggest that, in addition to other physiological roles, autophagy suppresses malignant transformation of normal cells, at least in part, through the degradation of oncogenic proteins, damaged mitochondria, and protein aggregates^16,17^. Following transformation, however, autophagy is conversely thought to promote malignant cell survival in response to stressors found in the tumor microenvironment (e.g., nutrient deprivation, and hypoxia), thus supporting tumor growth, invasion, and metastasis, as well as diminishing the effectiveness of chemo- and radiotherapies^18,19^. Whether autophagy functions similarly during human tumorigenesis is still under investigation; however, multiple clinical trials are underway to evaluate the efficacy of using the lysosomotropic alkalinizing agent, hydroxychloroquine, to sensitize tumors to chemotherapy^19^. Seemingly in support of the proposed role of autophagy in tumor survival, core *ATG* genes are generally not mutated or transcriptionally downregulated in most human cancers^20^. However, we have discovered that ATG5 is selectively inactivated in some human tumors by somatic mutations and aberrant mRNA splicing, as well as relative overexpression of *ATG16L2*, a heretofore unrecognized dominant-negative inhibitor of the ATG12–ATG5-ATG16L1 complex. Collectively, these perturbations disrupt a critical ATG5-ATG16L1 interaction and elicit proteasome-dependent degradation of ATG5, ATG12, and ATG16L1. Mechanistically, we have determined that an initial ATG5-ATG16L1 interaction is normally required to prevent the ubiquitin conjugation of ATG5 and instead facilitate its conjugation to ATG12, which in turn enhances its affinity for ATG16L1 and further stabilizes the ATG12–ATG5-ATG16L1 complex. Thus, the competing ubiquitin and ATG12 conjugation reactions naturally form a molecular “*conjugation switch*” that integrates protein quality control (PQC) of “orphaned” complex subunits with their assembly into ATG12–ATG5-ATG16L1 complexes. Dysregulation of this conjugation switch through multiple mechanisms in human tumors highlights the importance of ATG5 as a critical regulator of autophagy, and is consistent with the purported role of autophagy as a suppressor of tumor initiation.

## Results

### An *ATG5* splice site mutation in DU145 cells results in the loss of ATG5 expression and triggers proteasomal degradation of ATG12 and ATG16L1

**Fig. 1:**
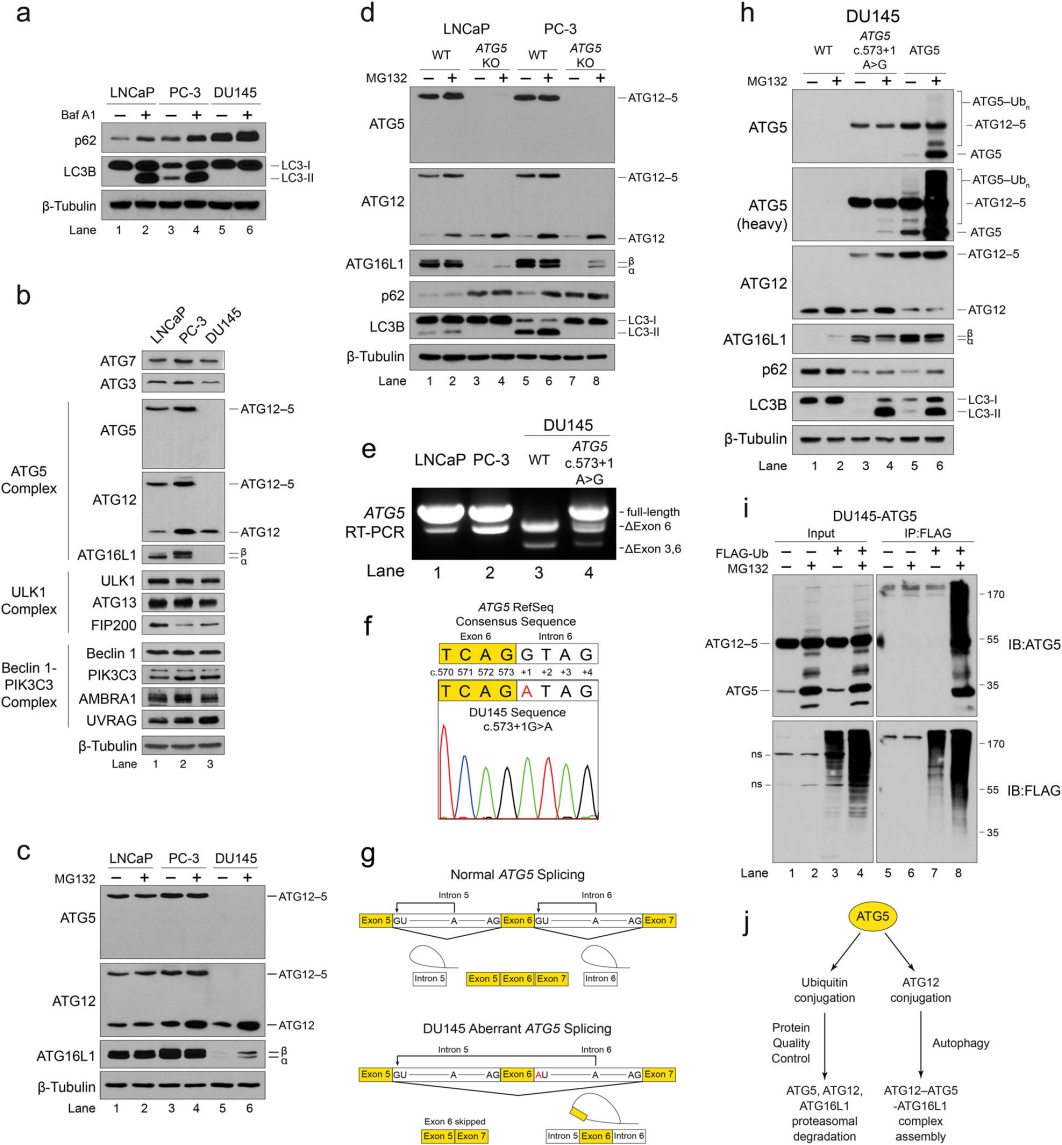
An *ATG5* splice site mutation in DU145 cells results in the loss of ATG5 expression and triggers proteasomal degradation of ATG12 and ATG16L1. **a** LNCaP, PC-3, and DU145 PCa cells were treated with 125 nM Bafilomycin A1 (Baf A1) for 8 h, and lysates were immunoblotted for markers of autophagosome formation (LC3B lipidation) and autophagic flux (p62 degradation). **b** Lysates from LNCaP, PC-3, and DU145 PCa cells were immunoblotted for components of the ATG5, ULK1, and Beclin 1-PIK3C3 complexes. **c** LNCaP, PC-3, and DU145 PCa cells were treated with 10 μM MG132 for 8 h, and immunoblotted for the indicated proteins. **d** LNCaP and PC-3 *ATG5* CRISPR/Cas9 knockout (*ATG5* KO) cell lines were treated with 10 μM MG132 for 8 h, and immunoblotted for the indicated proteins. **e** RT-PCR analysis of *ATG5* mRNA expression in wild-type LNCaP, PC-3, and DU145 cells, as well as DU145 cells possessing an *ATG5* c.573+1A>G splice site knock-in mutation. **f** Sequencing chromatogram of the *ATG5* exon 6/intron 6 boundary region in DU145 cells, compared to the consensus reference sequence. **g** Diagram of normal and aberrant *ATG5* splicing resulting from a splice donor site mutation in intron 6. **h** Wild-type DU145 cells, as well as those possessing an *ATG5* c.573+1A>G splice donor site knock-in mutation or stably expressing ectopic ATG5, were treated with 10 μM MG132 for 12 h and immunoblotted for the indicated proteins. **i** FLAG-ubiquitin was immunoprecipitated from DU145 cells stably expressing ectopic ATG5 and treated with 10 μM MG132 for 8 h. **j** Diagram of the ATG5-conjugation switch model. See also Supplementary Fig. S1

While assessing basal autophagic flux in classical prostate cancer (PCa) cell lines, we found that DU145 cells had strikingly higher basal levels of p62 compared to LNCaP and PC-3 cells (Fig. 1a). Moreover, inhibition of autophagic flux with Bafilomycin A1 (Baf A1) treatment triggered a significant build-up of lipid-conjugated LC3B (LC3B-II) in both LNCaP and PC-3 cells, whereas no LC3B-II was detected in DU145 cells (Fig. 1a). Expression levels of ULK1 and Beclin 1-phosphatidylinositol 3-kinase catalytic subunit type 3 (PIK3C3) complex subunits were comparable between LNCaP, PC-3, and DU145 cells; however, subunits of the ATG12–ATG5-ATG16L1 complex were expressed at lower levels in LNCaP cells, and were entirely absent in DU145 cells (Fig. 1b). Treatment of DU145 cells with the proteasome inhibitor, MG132, had no effect on ATG5 expression, but triggered a buildup of both ATG16L1 and unconjugated ATG12 (Fig. 1c, lanes 5 and 6), suggesting that DU145 cells did not express ATG5, and that orphaned ATG12 and ATG16L1 underwent proteasomal degradation.

To determine whether ATG12 and ATG16L1 stabilities were dependent upon ATG5, we knocked out *ATG5* in LNCaP and PC-3 cells using CRISPR/Cas9, which led to the loss of both ATG12 and ATG16L1 expression (Fig. 1d, lanes 3 and 7). Consistent with the previously reported compensatory induction of autophagy in response to proteasome inhibition^21^, MG132 treatment triggered an increase in LC3B-II levels in wild-type LNCaP and PC-3 cells (Fig. 1d, lanes 1, 2, 5, and 6), whereas no LC3B-II was detected in *ATG5* KO cells (Fig. 1d, lanes 3, 4, 7, and 8). MG132 treatment did, however, partially rescue ATG16L1 and unconjugated ATG12 expression in *ATG5* KO cells (Fig. 1d, lanes 4 and 8). ATG12 and ATG16L1 were similarly lost in *Atg*5^−/−^ mouse embryonic fibroblasts (MEFs) (Supplementary Fig. S1, lane 3), confirming that ATG12 and ATG16L1 stabilities were dependent upon ATG5 in multiple cell types.

In an effort to understand why DU145 cells lacked ATG5, we noted a recent study in which DU145 cells were found to undergo extensive alternative mRNA splicing for unknown reasons^22^. We confirmed the absence of full-length *ATG5* mRNA expression and, instead, found two splice variants that excluded either exon 6 or both exons 3 and 6 (Fig. 1e, lane 3). Since DU145 cells reportedly possess a hemizygous 6q deletion encompassing the *ATG5* locus^23^, and given that both mRNA variants lacked exon 6, we speculated that a splice site mutation proximate to exon 6 in the remaining allele might be responsible for “exon skipping” and the complete absence of full-length *ATG5* mRNA. We, therefore, sequenced the splice sites flanking exon 6 and discovered a mutation (c.573+1G>A) at the most 5′ nucleotide of intron 6 (Fig. 1f). This mutation affected the conserved splice donor site and was predicted to prevent nucleophilic attack by the 2′-hydroxyl group of the branch site adenosine, thereby triggering attack at the next upstream splice donor site of intron 5 and aberrant excision of exon 6 along with introns 5 and 6 (Fig. 1g). Remarkably, reverting the mutant splice site allele to wild type in DU145 cells using CRISPR/Cas9 rescued full-length *ATG5* mRNA expression (Fig. 1e, lane 4), as well as ATG12–ATG5 conjugation, functional ATG12–ATG5-ATG16L1 complex formation and autophagic flux, as determined by LC3 lipidation and p62 degradation (Fig. 1h, lanes 3 and 4). Collectively, these data proved that the autophagic defect in DU145 cells was caused by an *ATG5* splice donor site mutation, which resulted in the loss of ATG5 expression and proteasomal degradation of orphaned ATG12 and ATG16L1.

While reversion of the *ATG5* splice site mutation in DU145 cells restored the formation of ATG12–ATG5 conjugates, unconjugated ATG5 still remained undetectable (Fig. 1h, lane 3). Unconjugated ATG5 was faintly rescued by a 12 h incubation with MG132; however, this had no effect on conjugated ATG12–ATG5 (Fig. 1h, lane 4), suggesting that, like orphaned ATG12 and ATG16L1, free ATG5 also underwent proteasomal degradation in DU145 cells. Proteasomal degradation of free ATG5 is consistent with the fact that unconjugated ATG5 was also undetectable in wild-type LNCaP and PC-3 cells, as well as MEFs (Fig. 1b, lanes 1 and 2; Supplementary Fig. S1, lane 1). To confirm, we treated DU145 cells stably expressing ectopic ATG5 with MG132, which triggered a dramatic accumulation of ATG5 and a ladder of high-molecular weight species indicative of polyubiquitination (Fig. 1h, lanes 5 and 6). Similar results were obtained in *Atg5*^−/^^−^ MEFs stably expressing ectopic murine ATG5 (Supplementary Fig. S1, lanes 7 and 8). Co-immunoprecipitation of ATG5 using FLAG-tagged ubiquitin confirmed that free ATG5 was polyubiquitinated (Fig. 1i). Together, these data support the hypothesis that ATG5, ATG12, and ATG16L1 all undergo PQC when orphaned from the intact ATG12–ATG5-ATG16L1 complex. Since ATG5 is targeted by both ubiquitin and ATG12 conjugation reactions, we proposed a model in which the competing conjugation reactions effectively function as a molecular “conjugation switch” that regulates autophagy by promoting either the proteasomal degradation of ATG5, or its assembly into stable ATG12–ATG5-ATG16L1 complexes (Fig. 1j).

### Somatic *ATG5* splice site mutations and alternative mRNA splicing prevent ATG12 conjugation and trigger PQC of unstable protein isoforms

**Fig. 2:**
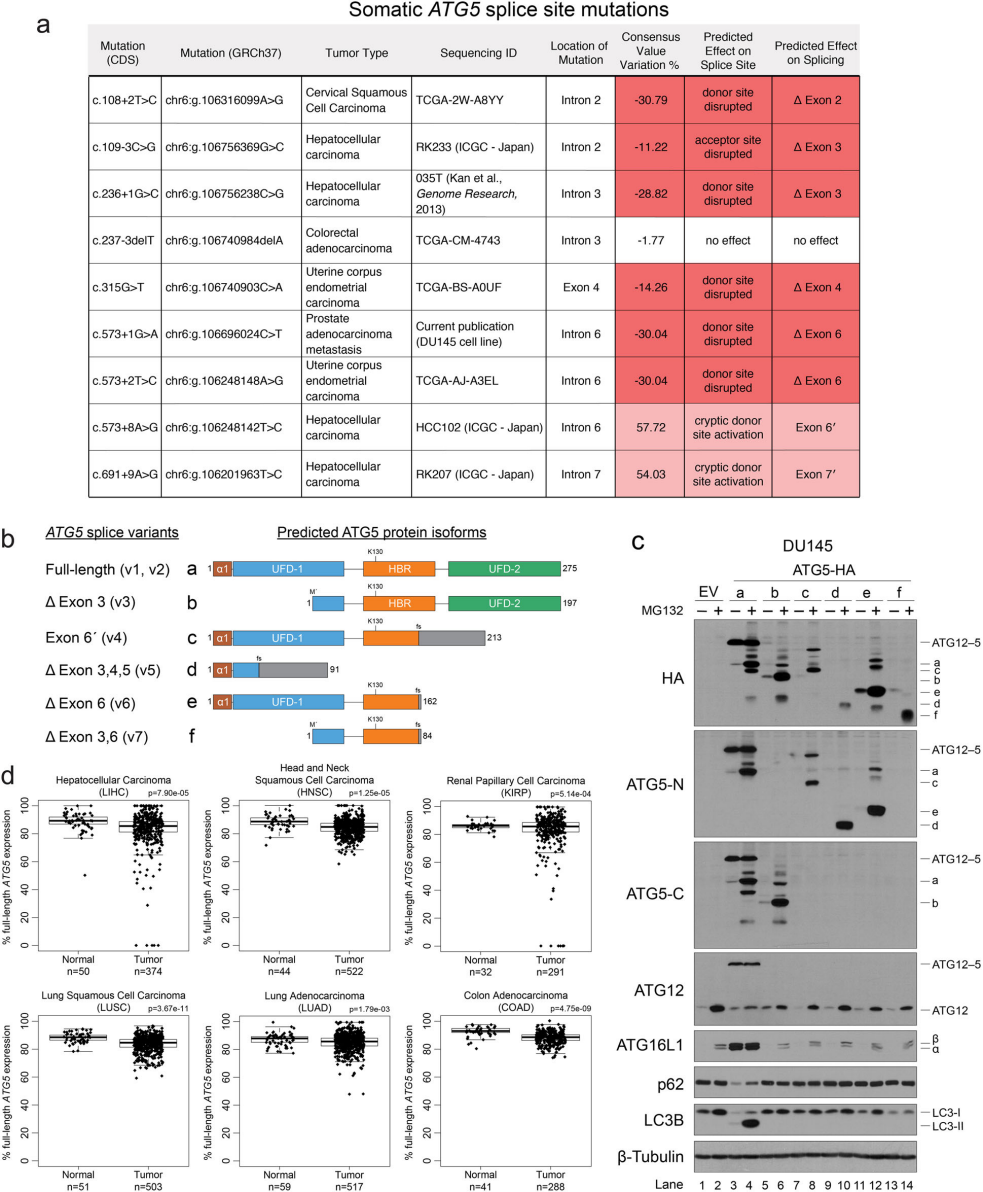
Somatic *ATG5* splice mutations and alternative mRNA splicing prevent ATG12 conjugation and trigger PQC of highly unstable protein isoforms. **a** Table of somatic *ATG5* splice site mutations identified in human tumor samples and cancer cell lines. The specific DNA mutations are indicated based on the coding sequence (CDS) and the genomic sequence (GRCh37). The location of the mutations within splice acceptor or donor sites of specific introns/exons is shown, as is the tumor type and sequencing ID of the tumors in which the mutations were identified. Finally, the effect of splice site mutations on *ATG5* splicing was predicted, in silico, using the Human Splicing Finder (http://www.umd.be/HSF/)^24^. The consensus value (CV) variation % is the difference between wild-type and mutant CVs as a percentage of the total wild-type CV. CVs are calculated based on positional variation from the consensus splicing motif. Predicted skipped exons are indicated with (Δ) and predicted cryptic exons are indicated with (′). **b** Diagram of predicted isoforms encoded by known *ATG5* splice variants. UFD-1 and UFD-2 refer to the ubiquitin-fold domains; HBR refers to the helix-bundle region; and α1 refers to the alpha-1 helix domain^27^. **c** DU145 cells, stably expressing an empty vector (EV) or HA-tagged ATG5 alternative protein isoforms, were treated with 10 μM MG132 for 8 h and immunoblotted for the indicated proteins. ATG5-N and ATG5-C refer to antibodies raised to the N- or C-terminus of ATG5, respectively. **d** Tukey box plots indicating the percentage of full-length *ATG5* mRNA (*ATG5v1*; uc003prf) expression to total *ATG5* mRNA expression from normal and tumor tissues. See also Supplementary Fig. S2

Having identified an *ATG5* splice site mutation in DU145 PCa cells that resulted in the loss of ATG5, ATG12, and ATG16L1 protein expression and the inactivation of autophagy, we next questioned whether other tumors might possess similar splice site mutations that impair mRNA splicing of *ATG5*. We searched human tumor mutation databases and found several unique *ATG5* splice site mutations identified in multiple tumor types. All but one of these mutations were predicted, in silico^24^, to either destroy the consensus splice site and trigger exon skipping or activate a cryptic splice site that would introduce a frameshift and prevent expression of full-length *ATG5* (Fig. 2a). However, it remained unclear whether these predicted splicing defects would lead to the loss of functional ATG5 expression as we had observed in DU145 cells.

To test whether known *ATG5* mRNA splice variants, listed in the National Center for Biotechnology Information (NCBI) RefSeq database^25^, encoded functional protein isoforms (Fig. 2b; Supplementary Fig. S2a, b), we stably expressed HA-tagged versions of each predicted isoform in DU145 cells. Notably, only full-length ATG5 (isoform a) formed functional ATG12–ATG5-ATG16L1 complexes as determined by the rescue of conjugated ATG12–ATG5 and ATG16L1 expression, as well as LC3B lipidation and p62 degradation (Fig. 2c, lanes 3 and 4). All other isoforms were essentially undetectable in the absence of proteasomal inhibition, which caused a dramatic accumulation of their polyubiquitinated forms (Fig. 2c, lanes 5–14). Importantly, polyubiquitination and proteasomal degradation occurred with isoforms containing C-terminal truncations caused by frameshifts and premature stop codons (isoforms c–f), as well as those with N-terminal deletions, predicted to result from the introduction of alternative translation initiation sites (isoforms b and f) (Fig. 2b; Supplementary Fig. S2a, b). Thus, both the N-and C-termini of ATG5 were critical for its stability. Moreover, no single region of ATG5 was shared among all isoforms, suggesting that ATG5 may be polyubiquitinated by multiple E3 ubiquitin ligases that recognize nonoverlapping regions of the protein.

The fact that all ATG5 protein isoforms encoded by known splice variants failed to conjugate with ATG12—and instead underwent ubiquitin conjugation that targeted them for PQC—suggested that any somatic splice site mutation that disrupts normal *ATG5* mRNA splicing in tumors likely leads to a complete loss of ATG5 function. These findings also raised the possibility that, independently of splice site mutations, tumor cells may routinely utilize “conventional” alternative splicing of *ATG5* as a mechanism to reversibly regulate ATG12–ATG5-ATG16L1 complex formation and autophagy. As aberrant mRNA splicing frequently occurs in tumors^26^, we tested this hypothesis by comparing the percentage of full-length *ATG5* (*ATG5v1*) expression from total *ATG5* expression in normal and tumor samples within The Cancer Genome Atlas (TCGA) datasets possessing at least 15 normal samples. While full-length *ATG5v1* was the dominant species in all tumor types, we found a statistically significant reduction in the percentage of full-length *ATG5* expression in 6 out of 12 tumor types (*p* < 0.01; Fig. 2d). Prostate adenocarcinoma was the lone tumor type in which the average percentage of full-length *ATG5v1* expression was increased in tumors compared to normal tissue; however, there were also a number of outlier prostate tumor samples in which full-length *ATG5v1* expression was dramatically reduced or completely lost (Supplementary Fig. S2c). Collectively, these data suggest that ATG12–ATG5-ATG16L1 complex assembly is regulated in tumors by conventional alternative *ATG5* mRNA splicing, as well as aberrant splicing resulting from *ATG5* splice site mutations, both of which lead to the coordinated degradation of ATG5, ATG12, and ATG16L1.

### Somatic *ATG5* missense, nonsense, and deletion mutations trigger PQC by directly disrupting the ATG16L1-binding pocket in ATG5

The fact that every alternative ATG5 isoform failed to conjugate to ATG12 was somewhat surprising given that all but one retained Lys-130 and were theoretically capable of conjugating to ATG12 (Fig. 2b). It was, therefore, unclear if the turnover of these ATG5 isoforms resulted from inherent instability due to improper folding, their inability to conjugate to ATG12, or perhaps because the N and/or C-terminal deletions disrupted other interactions that were essential for ATG5 stability. In the ATG12 (∆N-terminus)–ATG5-ATG16L1 (N-terminus) crystal structure, N- and C-terminal residues of ATG5 are located at the ATG16L1-binding interface, as opposed to the ATG12-binding interface^27^. Consequently, we suspected that the stability of ATG5, and thus its ability to conjugate to ATG12, might depend upon its initial interaction with ATG16L1. Importantly, this prediction differed from the current model of ATG12–ATG5-ATG16L1 complex formation in which conjugated ATG12–ATG5, constitutively expressed in the cytoplasm, is proposed to be recruited to phagophores by membrane-bound ATG16L1^2,3^.

**Fig. 3:**
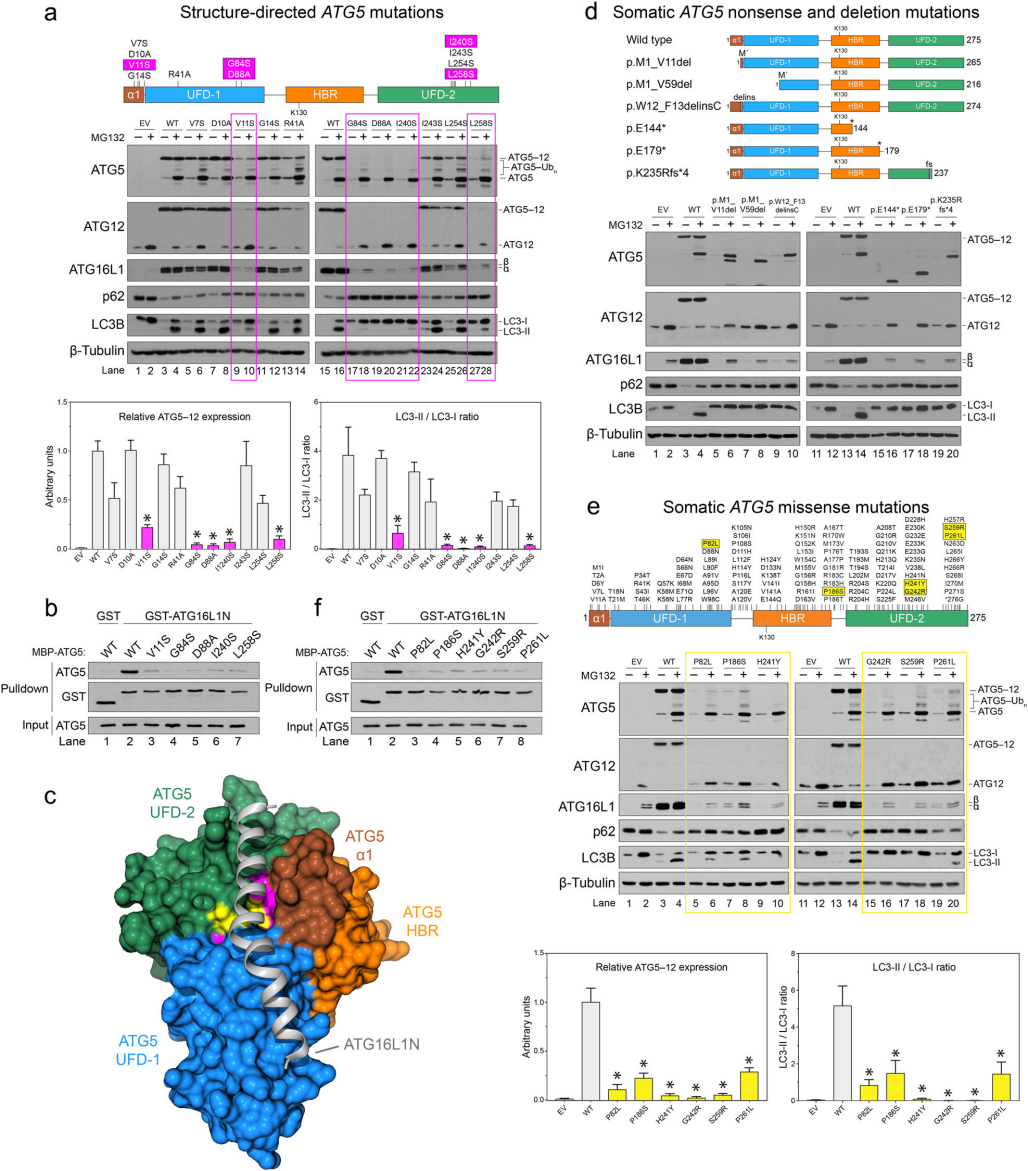
Somatic *ATG5* missense, nonsense, and deletion mutations trigger PQC by directly disrupting the ATG16L1-binding pocket of ATG5. **a** DU145 cells, stably expressing empty vector (EV), wild-type ATG5 (WT), or ATG5 mutants designed to disrupt the ATG16L1-binding region, were treated with 10 μM MG132 for 8 h and immunoblotted for the indicated proteins. Mutations that dramatically impair ATG12 and LC3B conjugation reactions are highlighted with magenta. The ATG12–ATG5 conjugate expression levels and LC3-II/LC3-I ratios from MG132 treated cells were quantified and graphed below (*, *p* < 0.01). **b** GST pull downs were performed using GST-ATG16L1N and recombinant wild-type or mutant ATG5. **c** Rendering of the ATG12ΔN–ATG5-ATG16L1N crystal structure (PDB ID: 4GDL). The ribbon backbone of ATG16L1N is depicted in gray. The surface rendering of ATG5 includes the α1-helix (brown), UFD-1 (blue), HBR (orange), and UFD-2 (green) domains. ATG12 is not visible in this orientation. Critical residues identified from targeted structural analyses are highlighted in magenta, while those arising from somatic missense mutations identified in tumors are highlighted in yellow. **d** Diagram of proteins predicted to result from *ATG5* nonsense and deletion mutations, and their fate upon expression in DU145 cells. **e** Diagram of *ATG5* missense mutations identified in human tumors and their fate upon expression in DU145 cells. The ATG12–ATG5 conjugate expression levels and LC3-II/LC3-I ratios from MG132 treated cells were quantified and graphed below (*, *p* < 0.01). Note the missense mutations (highlighted in yellow) that dramatically impair ATG12 conjugation and LC3B conjugation. **f** GST pull downs were performed using GST-ATG16L1N and recombinant wild-type or mutant ATG5. See also Supplementary Fig. S3 and Supplementary Table S1

To test this hypothesis, we used the ATG12∆N–ATG5-ATG16L1N crystal structure as a guide to introduce mutations that would be predicted to disrupt the ATG5-ATG16L1 interaction^27^. Stable expression of these ATG5 mutants in naturally deficient DU145 cells revealed several key residues (highlighted in magenta) that were essential for ATG5, ATG12, and ATG16L1 stability, ATG12 conjugation, and functional ATG12–ATG5-ATG16L1 complex formation, as determined by LC3B lipidation and p62 degradation (Fig. 3a). The most effective mutations (G84S, D88A, and I240S) led to complete degradation of the mutant protein, which fully prevented LC3B lipidation and caused an accumulation of p62 that was indicative of a block in autophagic flux (Fig. 3a, lanes 17–22). Using a glutathione *S*-transferase (GST) pull-down assay with bacterially expressed and purified GST-ATG16L1N and His-tagged wild-type and mutant ATG5 proteins, we confirmed that these mutations, along with the similarly effective mutations, V11S and L258S, disrupted the interaction of ATG16L1N with ATG5, in vitro (Fig. 3b). Mapping the loss-of-function mutations onto a surface rendering of the ATG12∆N–ATG5-ATG16L1N structure revealed a critical binding pocket where the N-terminal α1-helix and UFD-1 domains converged with the C-terminal UFD-2 domain (Fig. 3c, magenta). The fact that this ATG16L1-binding pocket was comprised of residues at both the N and C-terminal regions of ATG5 explained why all of the alternative protein isoforms containing N and/or C-terminal deletions were completely destabilized and failed to undergo ATG12 conjugation (Fig. 2b, c).

To determine if somatic mutations identified in human tumor samples directly affected this critical interaction between ATG5 and ATG16L1, we next compiled a list of all somatic *ATG5* coding-sequence mutations that have been identified across a multitude of human tumors and cancer cell lines (Supplementary Table S1). Notably, a recurrent frameshift mutation (c.704delA), predicted to cause a 38 amino acid C-terminal truncation (p.K235Rfs*4), has been identified in 17 unique tumors and cancer cell lines, including LNCaP PCa cells (Fig. 3d; Supplementary Table S1). Stable expression of this truncated mutant in DU145 cells, as well as five others predicted to arise from other *ATG5* nonsense or deletion mutations, failed to rescue the autophagy defect and resulted in complete degradation of the mutant proteins, along with ATG12 and ATG16L1 (Fig. 3d). Remarkably, even very small alterations to the N- or C-termini, including c.26_38delGTT (p.W12_F13delinsC), which affects only two amino acids within the N-terminal α1-helix domain, completely destabilized ATG5 (Fig. 3d, lanes 9 and 10). We, therefore, classified all somatic *ATG5* nonsense and deletion mutations affecting the N- or C-terminus as amorphic, loss-of-function mutations (Supplementary Table S1).

We also stably expressed more than 30 *ATG5* missense mutants in DU145 cells and found several (highlighted in yellow) that resulted in proteasomal degradation of ATG5, ATG12, and ATG16L1, and impaired or completely eliminated LC3B lipidation and p62 degradation (Fig. 3e; Supplementary Fig. S3a and b). H241Y, G242R, and S259R mutations were fully penetrant and thus were also classified as amorphic, loss-of-function mutations (Fig. 3e, lanes 9, 10, and 15–18; Supplementary Table S1). Interestingly, these residues were located directly adjacent to those we had determined were essential for ATG5 stability and ATG12 conjugation (Fig. 3c, note the proximity of yellow and magenta-colored residues). Since P82L, P186S, and P261L mutations all caused significant, albeit partial, impairment of ATG12 conjugation, we classified these as hypomorphic, partial loss-of-function mutations (Fig. 3e, lanes 5–8, 19, and 20; Supplementary Table S1). These proline residues are not found on the surface of ATG5, but localize deeper within the protein core and are therefore likely to be important for proper conformation of the critical N- and C-terminal domains. Regardless, as before, each of these amorphic or hypomorphic mutants failed to bind ATG16L1 in vitro (Fig. 3f). Based on these results, we would expect other mutations that affect the ATG16L1-binding pocket, including p.R9_D10delinsH, p.D88N, p.P186T, and p.M246V (Supplementary Table S1), to similarly impair or completely block ATG12–ATG5-ATG16L1 complex formation and autophagy. Collectively, our functional analysis of somatic *ATG5* mutations in human tumors demonstrated that a single recurring somatic frameshift mutation (c.704delA), as well as a variety of other unique somatic mutations specifically disrupt the ATG16L1-binding pocket. This effectively “flips” the ATG5 conjugation switch from ATG12 to ubiquitin conjugation, and in turn triggers PQC of ATG5, ATG12, and ATG16L1.

### ATG12 conjugation to ATG5 enhances its affinity for ATG16L1 and stabilizes otherwise transient ATG5-ATG16L1 interactions

**Fig. 4:**
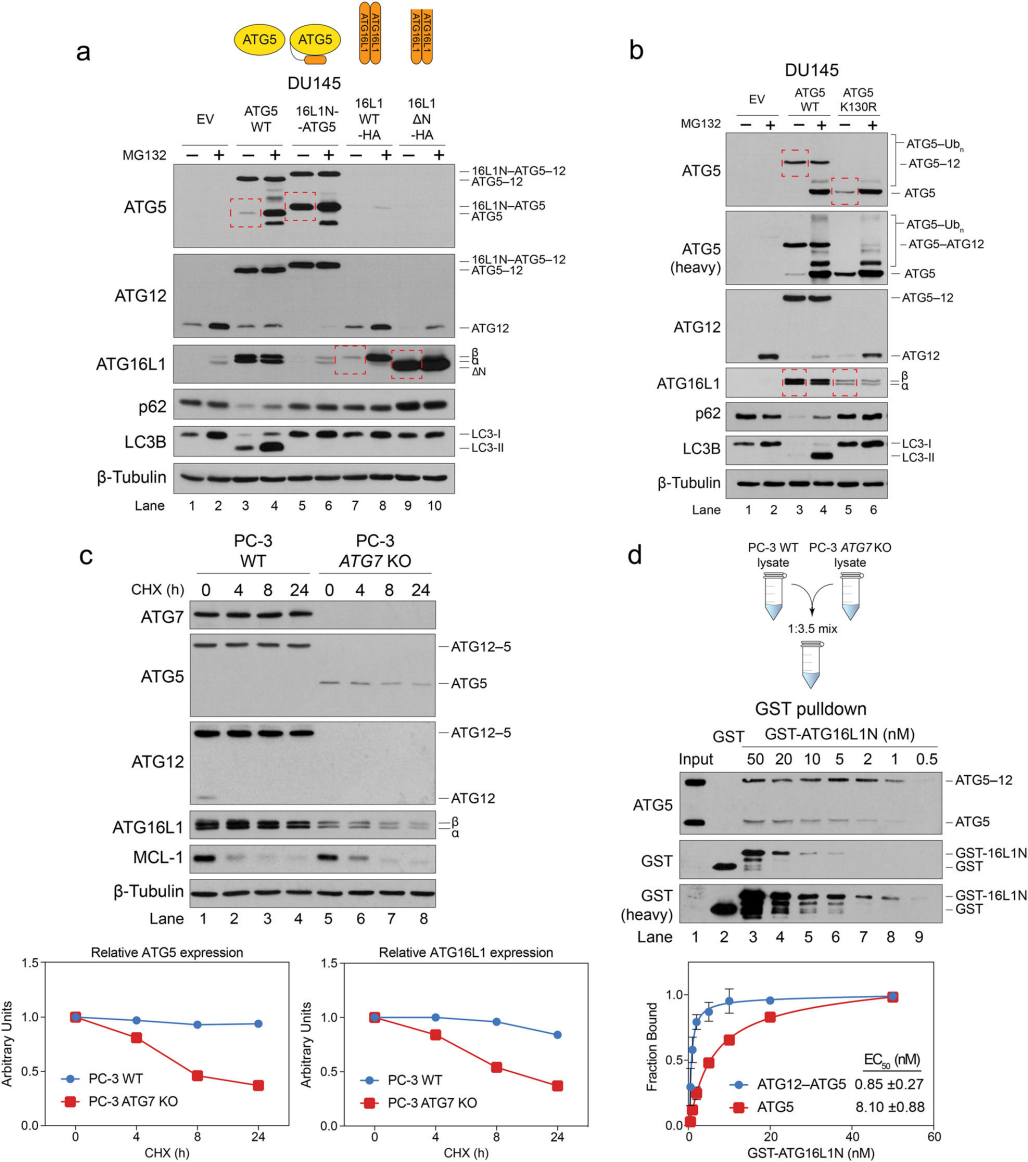
ATG12 conjugation to ATG5 enhances its affinity for ATG16L1 and stabilizes otherwise transient ATG5-ATG16L1 interactions. **a** DU145 cells, stably expressing empty vector (EV), wild-type ATG5 (WT), ATG5 tethered to an N-terminal fragment of ATG16L1 (16L1N-ATG5), wild-type ATG16L1, or an N-terminally deleted ATG16L1 (16L1ΔN), were treated with 10 μM MG132 for 8 h and immunoblotted for the indicated proteins. **b** DU145 cells, stably expressing EV, ATG5 WT, or a nonconjugatable ATG5 mutant (ATG5-K130R), were treated with 10 μM MG132 for 8 h and immunoblotted for the indicated proteins. **c** Wild-type and *ATG7* CRISPR/Cas9 knockout (*ATG7* KO) PC-3 cells were treated with 5 μg/mL cyclohexamide (CHX) for 4, 8, and 24 h and immunoblotted for the indicated proteins. Graphs were generated by normalizing each time point with its corresponding control (*t* = 0 h). **d** Lysates from wild-type (WT) and *ATG7* KO PC-3 cells were mixed in a 1:3.5 ratio to equalize the amount of conjugated to unconjugated ATG5. GST pull downs were then performed using the lysate mixture and recombinant GST or GST-ATG16L1N at the indicated concentrations. EC_50_ values for ATG12–ATG5 and ATG5 were determined using a best-fit curve of plotted values (fraction bound) from a total of six experiments. GST-ATG16L1N bound to native ATG12–ATG5 conjugate in lysates with ~tenfold higher affinity than endogenous ATG5. See also Supplementary Fig. S4

Contrary to the current model of ATG12–ATG5-ATG16L1 complex formation, in which ATG12 is conjugated to ATG5 prior to its recruitment to the phagophore by ATG16L1^2,3^, our characterization of *ATG5* mutations strongly suggested that the interaction of ATG5 with ATG16L1 was a prerequisite for ATG12 conjugation. To further evaluate this model, we stably expressed an ATG5 fusion protein in DU145 cells, in which an N-terminal fragment of ATG16L1 (aa 11–36), corresponding to its ATG5-binding domain, was tethered to ATG5 via a flexible linker (16L1N-ATG5). Remarkably, the tethered 16L1N fragment almost entirely prevented the ubiquitination and turnover of free ATG5 (Fig. 4a, compare unconjugated ATG5 in lanes 3 and 5). The tethered 16L1N fragment was even able to partially stabilize the otherwise unstable ATG5 (D88A) mutant and allow it to undergo ATG12 conjugation (Fig. 3a, lanes 19 and 20; Supplementary Fig. S4a, lanes 7 and 8), thereby confirming that mutation of the ATG16L1-binding pocket did not prevent ATG12 conjugation due to protein misfolding, etc. To ensure that binding of the tethered 16L1N fragment was responsible for the stabilization of ATG5, we also mutated the tethered 16L1N fragment (I17W), along with the ATG16L1-binding pocket in ATG5 (D88A), and found that disruption of this specific interaction resulted in complete degradation of the fusion protein (Supplementary Fig. S4a, compare 16L1N-ATG5 in lanes 5, 7, and 9).

In addition to stabilizing the ATG5 fusion protein and facilitating its conjugation to ATG12, the tethered 16L1N fragment also prevented the fusion protein from binding to and stabilizing endogenous ATG16L1, resulting in its proteasomal degradation and a block in LC3B lipidation and p62 degradation (Fig. 4a, lanes 3–6). This indicated that endogenous ATG16L1 possessed essential autophagic functions beyond simply stabilizing ATG5, which is consistent with the proposed role for its C-terminus in localizing the ATG12–ATG5-ATG16L1 complex to phagophores through its interactions with WIPI2 and/or FIP200^5–7^. Interestingly, we also stably expressed full-length ATG16L1 in DU145 cells, as well as a truncated ATG16L1 that lacked the N-terminal ATG5-binding domain (16L1ΔN), and found that, in the absence of ATG5, proteasomal degradation of ectopically expressed ATG16L1 was wholly prevented by removal of its N-terminus (Fig. 4a, lanes 7–10). Thus, in addition to binding ATG5 and protecting it from ubiquitination and proteasomal degradation, the N-terminus of ATG16L1 also functioned as a degron that targeted ATG16L1 for PQC when orphaned from ATG5.

While these findings suggested that ATG5 and ATG16L1 mutually stabilized and protected one another from PQC, they did not address the role of ATG12 conjugation on ATG5 or ATG16L1 stability. To investigate this, we stably expressed a K130R mutant of ATG5, which cannot undergo ATG12 conjugation, in DU145 cells and found that it was less stable than wild-type conjugated ATG5 and consequently stabilized ATG16L1 less efficiently (Fig. 4b, compare lanes 3 and 5). We also blocked ATG12 conjugation in LNCaP and PC-3 cells by knocking out *ATG7* using CRISPR/Cas9 and found that it substantially reduced ATG5 and ATG16L1 expression levels (Supplementary Fig. S4b, compare lanes 1, 3, 5, and 7). ATG5 and ATG16L1 levels were similarly reduced in *Atg7*^*−/*^^−^ and *Atg12*^*−/*^^−^ MEFs (Supplementary Fig. S4c, lanes 1–4). A cycloheximide chase experiment confirmed that free ATG5 (and ATG16L1) possessed dramatically shorter half-lives in *ATG7* KO PC-3 cells compared to conjugated ATG5 in wild-type cells (Fig. 4c). Together, these results suggested that the initial ATG5-ATG16L1 interaction was transient, perhaps due to lower affinity, or that the ATG12–ATG5 conjugate was resistant to ubiquitination.

The fact that ATG5-K130R was still readily polyubiquitinated and degraded (Fig. 4b, lanes 5 and 6) suggested that ATG12 conjugation did not stabilize ATG5 simply by blocking ubiquitination at Lys-130 (although, importantly, ubiquitination at Lys-130 would prevent conjugation of ATG12 to ATG5). Instead, since tethering of the ATG16L1N fragment to ATG5 was sufficient to fully stabilize unconjugated ATG5 (Fig. 4a, lane 5 and 6), we speculated that ATG12 conjugation induced a conformational change in ATG5 that increased its overall affinity for ATG16L1 and further stabilized the complex. Despite the high degree of structural similarity of ATG5 within the conjugated ATG12∆N–ATG5-ATG16L1N and unconjugated ATG5-ATG16L1N crystal structures (Supplementary Fig. S4d)^27,28^, we indeed found that conjugated ATG12–ATG5 possessed a roughly tenfold higher affinity for GST-ATG16L1N than did unconjugated ATG5 based on quantification of GST pull-down assays performed using cell lysates (Fig. 4d). While this result potentially explained the dramatic difference in stability between conjugated ATG12–ATG5 and unconjugated ATG5, it should be noted that it does not rule out the possibility that ATG12 conjugation may also directly impair ATG5 ubiquitination by sterically hindering the recruitment of E3 ubiquitin ligase(s) and/or access to key lysine residues on ATG5. It is also possible that additional unknown protein(s) associated with the native ATG12–ATG5-ATG16L1 complex (not present in the crystal structures) serve to enhance the interaction of ATG16L1 with conjugated ATG12–ATG5 and/or block E3 ligase recruitment.

### ATG16L2 functions as a dominant-negative inhibitor of autophagy by competing with ATG16L1 for binding to ATG5

**Fig. 5:**
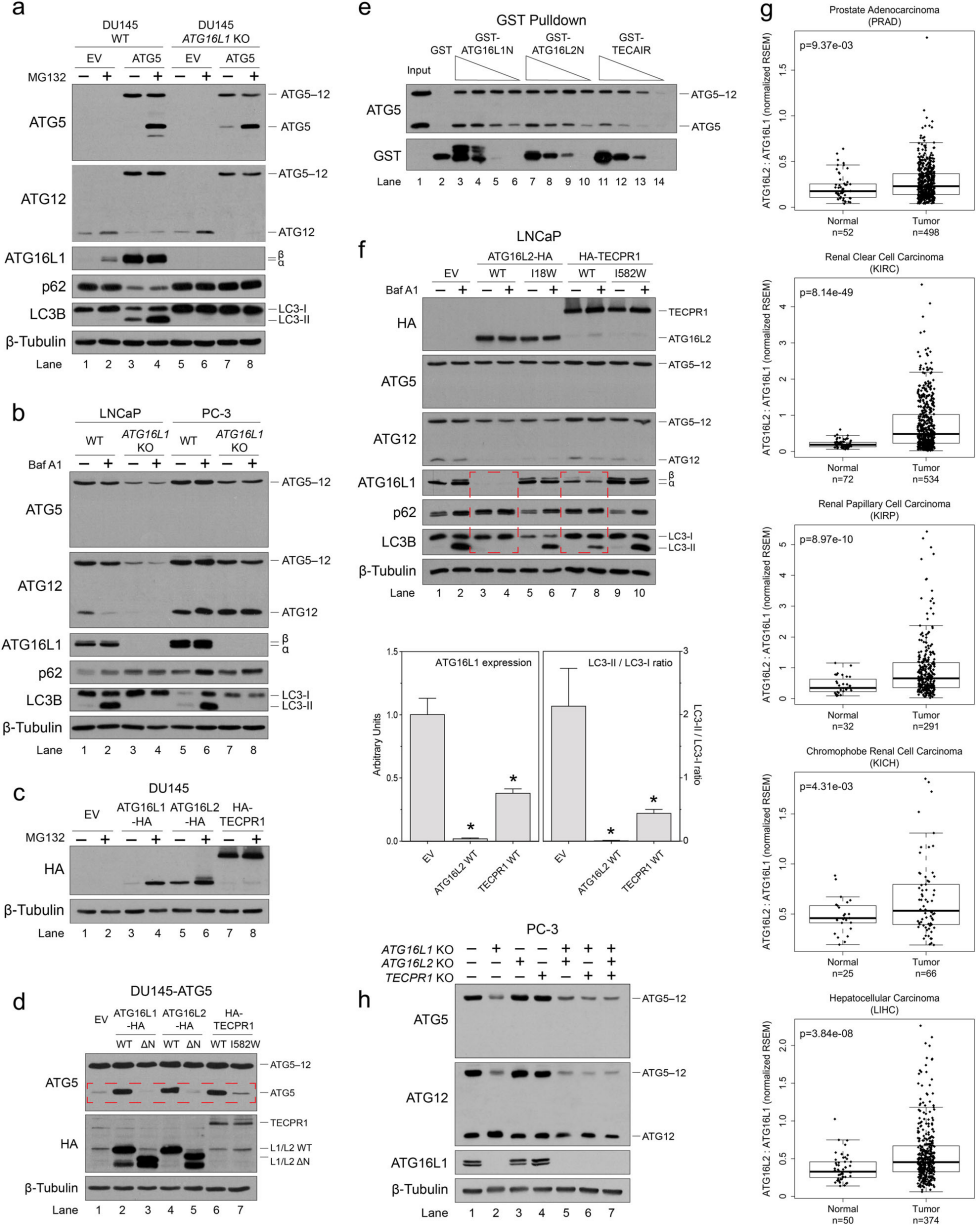
ATG16L2 functions as a dominant-negative inhibitor of autophagy by competing with ATG16L1 for binding to ATG5. **a** DU145 wild-type (WT) and *ATG16L1* CRISPR/Cas9 knockout cells (*ATG16L1* KO), stably expressing empty vector (EV) or ATG5, were treated with 10 μM MG132 for 8 h and immunoblotted for the indicated proteins. **b** LNCaP and PC-3 *ATG16L1* CRISPR/Cas9 knockout cell lines (*ATG16L1* KO) were treated with 125 nM Baf A1 for 8 h and immunoblotted for the indicated proteins. **c** DU145 cells, stably expressing an empty vector (EV), ATG16L1-HA, ATG16L2-HA, or HA-TECPR1, were treated with 10 μM MG132 for 8 h and immunoblotted with HA and β-tubulin antibodies. **d** Lysates from DU145 cells, stably co-expressing ATG5 and either ATG16L1-HA, ATG16L2-HA, HA-TECPR1, or their corresponding binding mutants (ΔN or I528W) were immunoblotted for ATG5, HA and β-tubulin. **e** Lysates from wild-type (WT) and *ATG7* KO PC-3 cells were mixed in a 1:3.5 ratio to normalize the amount of conjugated to unconjugated ATG5. As in Fig. 4d, GST pull downs were performed using the lysate mixture with recombinant GST (control), GST-ATG16L1N, GST-ATG16L2N, or GST-TECAIR (2–200 nM). **f** LNCaP cells, stably expressing wild-type (WT) ATG16L2-HA, HA-TECPR1, or their ATG5-binding mutants I18W and I152W, respectively, were treated with 125 nM Baf A1 for 8 h and immunoblotted for the indicated proteins. The ATG16L1 expression levels and LC3-II/LC3-I ratios from Baf A1-treated cells were quantified and graphed below (*, *p* < 0.01). **g** *ATG16L2:ATG16L1* mRNA ratios were determined for multiple tumor types from TCGA datasets. **h** Lysates from wild-type, *ATG16L1* KO, *ATG16L2* KO, *TECPR1* KO, *ATG16L1*/*L2* DKO, *ATG16L1*/*TECPR1* DKO, and *ATG16L1*/*L2*/*TECPR1* TKO PC-3 cells were immunoblotted for subunits of the ATG12–ATG5-ATG16L1 complex (see also Supplementary Fig. S5b, c)

Having determined that disruption of the ATG16L1-binding pocket in ATG5, through somatic mutations and/or alternative mRNA splicing, prevented ATG12 conjugation and formation of functional ATG12–ATG5-ATG16L1 complexes (Figs. 2 and 3), we expected that deletion of *ATG16L1* would similarly block both ATG12 and LC3 conjugation reactions. However, to our surprise, while knocking out *ATG16L1* in DU145 cells stably expressing ATG5 completely blocked LC3B lipidation and p62 degradation, ATG12 conjugation still occurred, albeit at reduced levels (Fig. 5a). ATG12–ATG5 conjugation was similarly impaired, but still present, in *ATG16L1* KO LNCaP and PC-3 cells (Fig. 5b). Finally, ATG12 conjugation was also observed in *Atg16l1*^*Δ/Δ*^ MEFs (Supplementary Fig. S4c, lanes 7 and 8).

In an attempt to resolve the apparent discrepancy between the ATG5 mutants that failed to bind ATG16L1, resulting in complete turnover of the ATG12–ATG5-ATG16L1 complex (Fig. 3), versus the *ATG16L1*-knockout data, wherein ATG12–ATG5 levels were only partially reduced (Fig. 5a, b), we postulated that ATG16L1-independent ATG12 conjugation might have occurred as a result of compensation from other ATG5-interacting proteins. ATG16L2 and TECPR1 reportedly bind to the same ATG16L1-binding pocket^28,29^, and thus might have similarly protected ATG5 from proteasomal degradation in *ATG16L1* KO cells (Supplementary Fig. S5a). Indeed, while ATG16L1 underwent complete PQC when expressed in ATG5-deficient DU145 cells, ATG16L2 and TECPR1 were both considerably more stable (Fig. 5c). Stably co-expressing ATG16L1, ATG16L2, or TECPR1 along with ATG5 in DU145 cells resulted in dramatic stabilization of unconjugated ATG5 (Fig. 5d, top panel, compare lanes 1, 2, 4, and 6). This stabilization was completely reversed by either deleting or mutating the ATG5-binding domains in each protein (Fig. 5d, lanes 3, 5, and 7), confirming that multiple proteins are capable of binding unconjugated ATG5 and preventing ubiquitination and proteasomal degradation. We then compared the relative affinities of GST-tagged ATG16L1N, ATG16L2N, and the **TEC**PR1 **A**TG5-**i**nteracting **r**egion (TECAIR) region of TECPR1^28^, for both conjugated ATG12–ATG5 and unconjugated ATG5 using GST pull-down assays. In agreement with previous biochemical characterizations of ATG16L2 and TECPR1^28,29^, we did not observe obvious differences in the affinities of GST-ATG16L1N and GST-ATG16L2N for either ATG5 or ATG12–ATG5, whereas GST-TECAIR displayed lower affinities for both at pH 8.0 (Fig. 5e). However, as we observed with GST-ATG16L1N (Fig. 4d; Fig. 5e, lanes 3–6), GST-ATG16L2N and GST-TECAIR both possessed higher affinities for conjugated ATG12–ATG5 compared to unconjugated ATG5 (Fig. 5e, lanes 7–14), indicating that the ATG12–ATG5-ATG16L2 and ATG12–ATG5-TECPR1 complexes were stabilized by ATG12 conjugation in a manner similar to that observed for the ATG12–ATG5-ATG16L1 complex.

Given these results, we then questioned whether ATG16L2 or TECPR1, which are not thought to catalyze LC3 lipidation^28,29^, might act as dominant-negative inhibitors of autophagy by competing with ATG16L1 for binding to ATG5 (Supplementary Fig. S5a). Stable overexpression of ATG16L2 or TECPR1 in wild-type LNCaP cells, which express lower levels of the ATG12–ATG5-ATG16L1 complex due to a hemizygous loss-of-function *ATG5* (c.704delA) mutation (Figs. 1b, 3d; Supplementary Table S1), triggered the displacement of endogenous ATG16L1 from ATG5, resulting in a significant loss of ATG16L1 and the inhibition of autophagy, as determined by a decrease in LC3 lipidation, with minimal effect on ATG12–ATG5 conjugation (Fig. 5f, lanes 3, 4, 7, and 8). Mutating or deleting the ATG5-binding domains of ATG16L2 and TECPR1 reversed these effects (Fig. 5f, lanes 5, 6, 9, and 10). Consistent with its higher affinity for ATG5 (Fig. 5e), ATG16L2 was more effective than TECPR1 at displacing ATG16L1 and inhibiting LC3B lipidation (Fig. 5f, lanes 3, 4, 7, and 8). Since either upregulation of ATG16L2 expression or downregulation of ATG16L1 expression in human tumors should increase the competitive binding of ATG16L2 to ATG5, we calculated the *ATG16L2*:*ATG16L1* mRNA expression ratio in normal and tumor samples from the TCGA mRNA datasets. The *ATG16L2:ATG16L1* ratios in the vast majority of normal samples were <1.0, suggesting that ATG16L1 was generally more highly expressed than ATG16L2. However, statistically significant increases in *ATG16L2* expression were observed, relative to *ATG16L1*, in 5 out of the 12 tumor types analyzed (*p* < 0.01; Fig. 5g). Together, these data indicated that ATG16L2—and to a lesser degree TECPR1—can act as dominant-negative inhibitors of autophagy by competitively binding to ATG5 and triggering PQC of the displaced ATG16L1. Overexpression of *ATG16L2*, therefore represents yet another mechanism for selectively impairing ATG12–ATG5-ATG16L1 complex assembly and autophagy in tumors.

To evaluate the role of endogenous ATG16L2 and TECPR1 in ATG12–ATG5-ATG16L1 complex formation, we knocked out *ATG16L2* and *TECPR1* in PC-3 cells using CRISPR/Cas9. Due to the lack of specific antibodies for these proteins, we verified knockout efficiency using Sanger sequencing and the Inference of CRISPR Edits (ICE) Analysis Tool (Supplementary Fig. S5b and c). Unlike the knockout of *ATG16L1*, which dramatically reduced ATG12–ATG5 levels, knockout of *ATG16L2* or *TECPR1* alone in PC-3 cells had no effect on ATG12–ATG5 conjugation (Fig. 5h, lanes 1–4). Given the similar affinities of ATG16L1 and ATG16L2 for ATG5 (Fig. 5e)^28,29^, this suggested that ATG16L2 was likely expressed at lower levels than ATG16L1. However, knockout of *ATG16L2* and *TECPR1*, in combination with *ATG16L1*, further reduced ATG12 conjugation compared to the knockout of *ATG16L1* alone, although it did not eliminate it entirely (Fig. 5h, lanes 2 and 7).

Since the ATG16L1-binding pocket of ATG5 must be occupied to prevent ubiquitin conjugation and PQC of ATG5, ATG12, and ATG16L1, as observed with ATG16L1-binding mutants (Fig. 3), the residual ATG12–ATG5 conjugation in the triple-KO cells suggested that other unknown protein(s) were able to bind free ATG5 and partially compensate for the concurrent loss of *ATG16L1*, *ATG16L2*, and *TECPR1*. Therefore, the data indicate that ATG16L2, TECPR1, and likely other unknown ATG5-binding protein(s), compete with ATG16L1 for binding to ATG5 and collectively function as dominant-negative inhibitors of ATG12–ATG5-ATG16L1 complex formation. It is imperative to reiterate that disruption of the ATG16L1-binding pocket in tumors through somatic *ATG5* mutations and/or alternative mRNA splicing, simultaneously prevents all proteins from binding to the ATG16L1-binding pocket, thus highlighting its critical importance in the regulation of the ATG5-conjugation switch, ATG12–ATG5-ATG16L1 complex formation and autophagy in tumors.

## Discussion

**Fig. 6:**
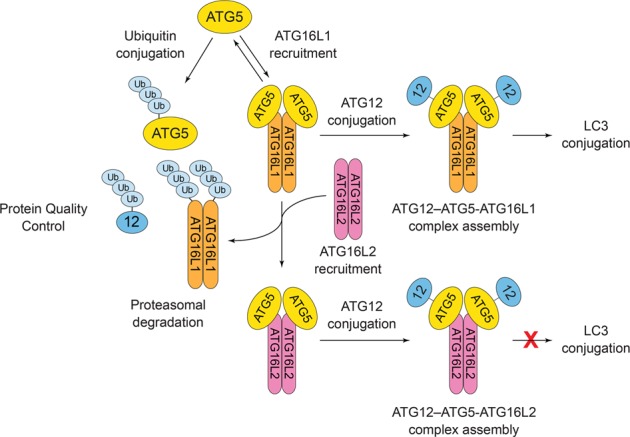
Revised model for ATG12–ATG5-ATG16L1 complex formation. A “*conjugation switch*” targeting ATG5 integrates protein quality control (PQC) and ATG12–ATG5-ATG16L1 complex assembly. Free ATG5, ATG12, and ATG16L1 are inherently unstable and are targeted by PQC until ATG5 transiently associates through a lower affinity interaction with ATG16L1, which impairs ubiquitin conjugation and facilitates ATG12 conjugation. ATG12 conjugation in turn enhances the affinity of ATG5 for ATG16L1, forming a stable ATG12–ATG5-ATG16L1 complex that catalyzes LC3 lipidation. ATG16L2 (and to a lesser degree TECPR1) hijacks the switch and acts as an endogenous dominant-negative inhibitor of ATG12–ATG5-ATG16L1 complex formation by competing with ATG16L1 for binding to ATG5, leading to its proteasomal degradation and the inhibition of LC3 lipidation and autophagy

This study was initiated following our discovery that DU145 PCa cells did not express ATG5 due to an *ATG5* splice donor site mutation, which ultimately triggered PQC of orphaned ATG12 and ATG16L1 and inactivated autophagy. We then identified and characterized *ATG5* splice variants and more than 50 structure-guided and somatic cancer mutations, which revealed that the mutually stabilizing ATG5-ATG16L1 interaction was an essential prerequisite for ATG12 conjugation and ATG12–ATG5-ATG16L1 complex formation. Due to the unique structure of ATG5 in which both the N- and C-termini collectively form the ATG16L1-binding pocket, somatic mutations and alternative/aberrant mRNA splicing affecting either region of the protein triggered complete proteasomal degradation of ATG5, and consequently ATG12 and ATG16L1 (Figs. 1, 3; Supplementary Table S1). Based on these findings we propose an updated model for ATG12–ATG5-ATG16L1 complex formation in which free, unbound ATG5, ATG12, and ATG16L1 are inherently highly unstable proteins that continuously undergo PQC until a transient, but essential, ATG5-ATG16L1 interaction temporarily impairs ubiquitin conjugation of ATG5, thereby stabilizing both proteins and allowing for ATG12 conjugation to ATG5 (Fig. 6). This initial lower affinity interaction between ATG5 and ATG16L1 likely stabilizes both proteins by masking E3 ubiquitin ligase binding sites and/or target lysine residues. Regardless, once formed, the ATG12–ATG5 conjugate displays a significantly enhanced affinity for ATG16L1, resulting in the formation of more stable ATG12–ATG5-ATG16L1 complexes. Therefore, by controlling the fate of ATG5, the competing ATG12 and ubiquitin conjugation reactions effectively function as a molecular “conjugation switch” that regulates autophagy by integrating PQC of individual subunits with ATG12–ATG5-ATG16L1 complex formation.

In addition to ATG16L1, we determined that ATG16L2—and to a lesser extent TECPR1—can hijack this conjugation switch by competitively binding to the ATG16L1-binding pocket of ATG5, displacing ATG16L1, and triggering its proteasomal degradation (Fig. 6). While the ATG12–ATG5-ATG16L2 complex has no known function, the ATG12–ATG5-TECPR1 complex reportedly mediates selective autophagy of bacterial pathogens (xenophagy) and participates in autophagosome–lysosome fusion^30,31^. Thus, while these complexes may indeed have important downstream functions in autophagy, neither catalyzes LC3 lipidation, which allows them to function as endogenous dominant-negative inhibitors of ATG12–ATG5-ATG16L1 complex assembly and canonical autophagy.

The fact that *ATG5* is selectively inactivated by somatic mutations and/or alternative mRNA splicing (Figs. 1 and 3), and that *ATG16L2* is transcriptionally overexpressed, relative to *ATG16L1*, in multiple tumor types (Fig. 5g), suggests that the inhibition of autophagy is indeed oncogenic. This is consistent with the current consensus that autophagy maintains homeostasis in pre-malignant cells and suppresses tumor initiation by eliminating injured mitochondria, oncogenic proteins and protein aggregates^16,17^. However, autophagy is also thought to be essential for tumor progression, metastasis and therapy resistance by promoting tumor cell survival during times of acute stress, e.g., in the hypoxic, oxidative, and nutrient-deprived tumor microenvironment prior to angiogenesis or in response to therapy^32–34^. Therefore, one would expect tumors possessing *ATG5* loss-of-function mutations or overexpressing *ATG16L2* to be deficient in their abilities to progress, metastasize, and/or develop resistance to chemo- or radiotherapies. If true, small molecule “ATG16L2-mimetics” that target the critical ATG16L1-binding pocket of ATG5 and trigger PQC of ATG16L1 could represent a new class of highly selective autophagy inhibitors that could be used therapeutically for autophagy-proficient tumors, particularly those with low-ATG5 expression resulting from transcriptional repression, alternative mRNA splicing or hemizygous somatic mutations.

This possibility notwithstanding, the fact that DU145 PCa cells, which were originally isolated from a brain metastasis^35^, are ATG5-deficient and readily form tumors in immunodeficient mice, suggests that ATG5 per se may not always be essential for tumor progression. The deletion of *Atg5* or *Atg7* has reportedly opposing effects on tumor progression, depending upon the status of *Tp53* or *Pten*, in murine pancreatic tumor models^36,37^. Thus, inactivation of autophagy may actually be tumor promotive in some genetic contexts, possibly due to reduced autophagy-dependent tumor antigen presentation and tumor immunosurveillance^38,39^. It is also possible that, once initiated, certain human tumors evolve the ability to utilize an ATG5-independent form of autophagy that enables growth and progression. Evidence for ATG5-independent autophagy has been reported in ATG5-deficient cells in response to specific types of stress and during the induction of pluripotency^40–43^.

Another intriguing possibility is that ATG5 may possess tumor suppressive functions that are unrelated to autophagy so that the loss of ATG5 (or the subsequent degradation of ATG12 and ATG16L1 via PQC), confers a selective advantage to rapidly dividing or migrating tumor cells, despite the loss of canonical autophagy. ATG5 reportedly mediates apoptosis and mitotic catastrophe through interactions with BCL-X_L_ and survivin, respectively^44,45^. Recently, unconjugated ATG12, which can induce apoptosis by binding to and inactivating anti-apoptotic BCL-2 family members^46^, was shown to be downregulated through ubiquitination and proteasomal degradation in cancer cells possessing RAS mutations^47,48^. While it is unknown if ATG16L1 likewise possesses pro-apoptotic activity, hemizygous *ATG5* deletions and *ATG5* mRNA downregulation in human melanomas are associated with metastasis and poor patient survival^49,50^. Thus, in order to effectively evaluate the therapeutic potential of targeting the ATG16L1-binding pocket in ATG5, further investigation into the potential ATG5-independent forms of autophagy; the nonautophagic functions of ATG5, ATG12, and ATG16L1; and the impact of ATG5 inactivation on tumor progression in different tissues and genetic backgrounds will be required.

## Materials and methods

### Reagents

**Table:** 

Reagent	Source	Identifier
*Antibodies*		
AMBRA1 (1:500 dilution)	Cell Signaling Technology	Cat#12250; RRID: N/A
ATG3 (1:500 dilution)	Sigma-Aldrich	Cat#3415; RRID: AB_2059244
ATG5 (C-terminal) (1:500 dilution)	Cell Signaling Technology	Cat#2630; RRID: AB_2062340
ATG5 (N-terminal) (1:8000 dilution)	Novus Biologicals	Cat#NB110-53818; RRID: AB_828587
ATG7 (1:500 dilution)	Cell Signaling Technology	Cat#2631; RRID: AB_2227783
ATG12 (D88H11) (1:500 dilution)	Cell Signaling Technology	Cat#4180; RRID: AB_1903898
ATG13 (1:2000 dilution)	Cell Signaling Technology	Cat#6940; RRID: N/A
ATG16L1 (D6D5) (1:2000 dilution)	Cell Signaling Technology	Cat#8089; RRID: AB_10950320
Beclin 1 (1:1000 dilution)	Cell Signaling Technology	Cat#3738; RRID: AB_490837
β-Tubulin (1:20,000 dilution)	Developmental Studies Hybridoma Bank (DSHB)	Cat#E7-c; RRID: N/A
FIP200/RB1CC1 (1:4000 dilution)	Proteintech Group	Cat#17250-1-AP; RRID: AB_10666428
FLAG (M2) (1:32,000 dilution)	Sigma-Aldrich	Cat#F7425; RRID: AB_439687
GST (91G1) (1:20,000 dilution)	Cell Signaling Technology	Cat#2625; RRID: AB_490796
HA (1:8000 dilution)	Novus Biologicals	Cat#NB600-363; RRID: AB_10001504
IgG-peroxidase (mouse; 1:2000 dilution)	Sigma-Aldrich	Cat#A4416; RRID: AB_258167
IgG-peroxidase (rabbit; 1:2,0000 dilution)	Sigma-Aldrich	Cat#A4914; RRID: AB_258207
LC3B (1:2000 dilution)	Cell Signaling Technology	Cat#2775; RRID: AB_915950
MCL-1 (1:1000 dilution)	Cell Signaling Technology	Cat#4572; RRID: AB_2281980
PIK3C3/Vps34 (ZMD.350) (1:1000 dilution)	Innovative Research	Cat#38-2100; RRID: AB_431499
p62/SQSTM1 (1:2000 dilution)	Cell Signaling Technology	Cat#5114; RRID: AB_10624872
ULK1 (1:500 dilution)	Cell Signaling Technology	Cat#8054 RRID: AB_11178668
*Chemicals*		
Bafilomycin A1	Tocris Bioscience	Cat#1334; CAS: 88899-55-2
Cyclohexamide	Calbiochem	Cat#239763; CAS: 66-81-9
MG132	Selleck Chemicals	Cat#S2619; CAS: 133407-82-6
*Oligonucleotides*		
RT-PCR primer sequences	This paper	See Table S2
Genomic PCR primer sequences	This paper	See Table S3
sgRNA oligo sequences	This paper	See Table S4
ssODN donor template sequence	This paper	See Table S5
Cloning primer sequences	This paper	See Table S6
Site-directed mutagenesis primer sequences	This paper	See Table S7

### Expression constructs

The pLOC lentiviral expression vector was obtained from the University of Texas MD Anderson Cancer Center (UTMDACC) Functional Genomics Core (FGC). FLAG-tag and HA-tag sequences were cloned into the *Bam*HI/*Nhe*I of pLOC to generate an N-terminal FLAG-tag (pLOC-NFLAG) or HA-tag (pLOC-NHA) vectors. HA-tag was also cloned into the *Nhe*I/*Asc*I sites of pLOC to generate a C-terminal HA-tag (pLOC-HA). cDNA of ubiquitin was kindly provided by Dr. Colin Duckett (Duke University School of Medicine, Durham, NC). Ubiquitin was cloned into *Nhe*I/*Asc*I sites of pLOC-NFLAG. cDNA for human *ATG5* (Cat#PLOHS_10007) was obtained from UTMDACC FGC (Houston, TX). Full-length *ATG5* was cloned into *Bam*HI/*Nhe*I sites of untagged pLOC and pLOC-HA. For alternative splice variant *ATG5*v3, only the predicted coding sequence was amplified by PCR and cloned into *Bam*HI/*Nhe*I sites of pLOC-HA. *ATG5*v4 was generated by site-directed mutagenesis. *ATG5*v5, v6 and v7 splice variants were originally cloned into pGEM-T easy following reverse transcription polymerase chain reaction (RT-PCR) of mRNA extracted from DU145 and PC-3 PCa cells, and were then subcloned into *Bam*HI/*Nhe*I sites of pLOC-HA. For bacterial expression, full-length *ATG5* was cloned into *Bam*HI/*Not*I of malE-pET, a bacterial expression vector kindly provided by Dr. Hung-wen (Ben) Liu (University of Texas at Austin, Austin, TX) that was derived from pET28b and contains the malE gene encoding a Maltose-binding protein (MBP) tag. All *ATG5* mutations were introduced by site-directed mutagenesis, except for mutations predicted to cause alternative translation initiation and N-terminal deletions (p.M1_V11del and p.M1_V59del) in which case only the predicted coding sequences were cloned. cDNA for murine *Atg5* was a gift from Roberta Gottlieb (Addgene plasmid #13095)^51^. Murine *Atg5* was cloned into *Bam*HI/*Nhe*I sites of untagged pLOC.

To clone *ATG16L1*, cDNA was obtained from the UTMDACC FGC (Cat#PLOHS_10007). The internal *Bam*HI site of *ATG16L1* was first removed by site-directed mutagenesis. Full-length and N-terminally deleted (ΔN) *ATG16L1* (nucleotides 118–1824) were then amplified by PCR and cloned into the *Bam*HI/*Nhe*I sites of pLOC-HA. cDNAs for *ATG16L2* (Cat#HsCD00342734) and *TECPR1* (Cat#HsCD00337885) were obtained from the Dana-Farber/Harvard Cancer Center DNA Resource Core. *ATG16L2* and *TECPR1* were cloned into the *Bam*HI/*Nhe*I sites of pLOC-HA and the *Nhe*I/*Asc*I sites of pLOC-NHA, respectively. Mutations of *ATG16L2* and *TECPR1* were subsequently introduced by site-directed mutagenesis. The 16L1N-ATG5 fusion construct was created by cloning the ATG5-binding region of *ATG16L1* (nucleotides 31–108) into the *Bam*HI/*Nhe*I sites of pLOC, and a 15 base-pair GS-linker-*ATG5* into the *Nhe*I/*Asc*I sites. Mutations were once again introduced by site-directed mutagenesis. For bacterial expression vectors, nucleotides 1–207 of *ATG16L1* (ATG16L1N) and *ATG16L2* (ATG16L2N), as well as nucleotides 1696–1830 of *TECPR1* (TECAIR), were cloned into the *Bam*HI/*Not*I sites of pGEX-4T-1 (GE Healthcare Bio-Sciences #28-9545-49).

### Cell lines and culture conditions

DU145 (HTB-81), LNCaP clone FGC (CRL-1740), and PC-3 (CRL-1435) PCa cells were purchased from American Type Culture Collection (ATCC) and grown in RPMI-1640, supplemented with 10% fetal bovine serum (FBS) and 2 mM l-glutamine, at 37 °C in humidified air containing 5% CO_2_ and were routinely passaged every 3 days. *Atg5*^*+/+*^, Atg5^−/−^, *Atg7*^*+/+*^, *Atg7*^*−/*^^−^, *Atg12*^*+/+*^, *Atg12*^*−/−*^, *Atg16L1*^*+/+*^, and *Atg16l1*^*Δ/Δ*^ MEFs were kindly provided by Dr. Noburu Mizushima (University of Tokyo, Tokyo, JP)^52^, Dr. Masaaki Komatsu (Tokyo Metropolitan Institute of Medical Science, Tokyo, JP)^53^, Dr. Jayanta Debnath (University of California-San Francisco, San Francisco, CA)^54^, and Dr. Shizuo Akira (Osaka University, Osaka, JP)^55^, respectively. All MEFs, as well as human embryonic kidney cells (HEK293T) provided by Dr. Casey Wright (The University of Texas Medical Branch, Galveston, TX), were cultured in DMEM, supplemented with 10% FBS and 4mM l-glutamine, at 32.5–37 °C in humidified air containing 5% CO_2_ and were routinely passaged every 3 days.

### RT-PCR and genomic PCR

mRNA was isolated from LNCaP, PC-3, and DU145 PCa cells using an RNeasy Kit with on-column DNase digestion (Qiagen #74104). cDNA was synthesized from 1 μg of mRNA using SuperScript^™^ III First-Strand Synthesis SuperMix for qRT-PCR (Invitrogen #11752-050), and *ATG5* was then amplified by PCR. Genomic DNA was isolated using the Wizard^®^ Genomic DNA Purification Kit (Promega #A1120). Genomic PCR was performed using FailSafe^™^ PCR System (Lucigen #FS99100). All PCR mixtures were resolved by gel electrophoresis and, when applicable, the resulting bands were cut, extracted and Sanger sequenced.

### Lentiviral transduction and stable cell line generation

HEK293T cells were co-transfected with pLOC along with psPAX2 and pHCMV-G lentivirus packaging plasmids. psPAX2 was a gift from Dr. Didier Trono (Addgene plasmid #12260). Approximately, 48 h following transfection, the medium was collected and 6 μL of sterile hexadimethrine bromide (5 μg/μL; Sigma-Aldrich #H9268) was added. The collected medium was then filtered through a 0.45 μm polyvinylidene fluoride (PVDF) syringe filter and incubated with target cells overnight. Transduction efficiency was evaluated by GFP expression and/or immunoblotting.

### CRISPR/Cas9 knockout and knock-in cell line generation

For knockout cell lines, DNA oligonucleotides (oligos) containing gRNA target sequences for *ATG5*, *ATG7*, *ATG16L1*, *ATG16L2*, and *TECPR1* were designed using the CRISPR Design Tool (http://crispr.mit.edu). DNA oligos were annealed and ligated into the lentiCRISPRv2 vector, a gift from Dr. Feng Zhang (Addgene plasmid #52961), using an established protocol^56^. DU145, PC-3, or LNCaP PCa cells were then transduced as described above, and sorted into single cell clones by flow cytometery (BD Biosciences, FACSAria^™^ Fusion). The clones were screened for successful gene knockout by immunoblotting for the targeted protein, as well as for LC3B lipidation and p62 degradation. For ATG16L2 and TECPR1, no commercial antibodies were found to be suitable, therefore, all clones were screened by Sanger sequencing and analyzed using the ICE Analysis Tool (https://ice.synthego.com). As cancer cells are frequently polyploidal, CRISPR/Cas9-mediated insertions or deletions (indels) result in multiple unique alleles that make Sanger sequencing challenging. This tool uses an algorithm to deconvolute Sanger trace data into unique alleles, which are then given “contribution percentages” based on the relative heights of trace data. The ICE score refers to the percentage of total alleles possessing indels, while the KO score refers to the percentage of total alleles possessing indels predicted to induce frameshifts and loss of gene function (see https://www.biorxiv.org/content/biorxiv/early/2019/01/14/251082.full.pdf for details). Clones with >90% KO scores were considered true knock outs.

For the *ATG5* splice donor site knock-in cell line, DNA oligos containing a gRNA target sequence for exon 6 of *ATG5* were designed using the CRISPR Design Tool. DNA oligos were annealed and ligated into the lentiCRISPRv2 vector. A single-strand DNA oligonucleotide (ssODN) donor template was designed to introduce the desired splice donor site mutation (c.573+1A>G) and four additional silent mutations intended to prevent cleavage of the donor template. DU145 cells were transduced as described above and electroporated the following day with the ssODN donor template (10 μM) using the DU145 program of the 4D-Nucleofector^™^ System (Lonza). Cells were then sorted into single clones by flow cytometry as described above. Successful homologous recombination was determined by immunoblotting and sequencing.

### Immunoblot analysis

Cells were lysed in ice-cold radioimmunoprecipitation assay buffer (50 mM Tris-HCl pH 7.4, 150 mM NaCl, 1% NP-40, 1% Na-deoxycholate) with protease inhibitors. Lysate protein concentrations were quantified using the Bradford assay, and 50 μg of lysate was loaded into 10–15% polyacrylamide gels and separated at 125 V for 1.5 h. The resolved proteins were then transferred onto nitrocellulose membranes (or PVDF membranes for ATG12 and LC3B immunoblots) for 2 h at 90 V. Membranes were blocked in 5% nonfat milk in TBS-T (50 mM Tris, 150 mM NaCl, 0.1% Tween-20, pH 7.6) for 1 h at room temperature, washed twice in TBS-T for 5 min, and incubated in primary antibody overnight at 4 °C with constant agitation. Membranes were then washed and incubated for 1 h at room temperature with mouse or rabbit IgG-HRP secondary antibodies (Sigma-Aldrich #A4416 and #A4914) diluted 1:2000 with 5% milk in TBS-T. Finally, membranes were washed and developed using enhanced chemiluminescence (PerkinElmer #NEL104001). When applicable, densitometry was performed using Image Studio Lite (LI-COR Biosciences).

### Recombinant protein expression and GST pull downs

All forms of recombinant MBP-ATG5, as well as GST, GST-ATG16L1N, GST-ATG16L2N, and GST-TECAIR were expressed in *Escherichia coli* strain BL21(DE3)pLysS (EMD Millipore #694510) following an overnight induction at 18 °C with 1 mM isopropyl-β-d-thiogalactoside (IPTG). Recombinant proteins were then purified using fast protein liquid chromatography, coupled to a Ni^2+^-NTA column (Thermo Scientific #88222) or a glutathione column (EMD Millipore #70541). All proteins were dialyzed into phosphate-buffered saline (PBS) and concentrations were determined using the Bradford assay. For GST pull-down assays of recombinant MBP-ATG5 mutants, 200 nM GST-ATG16L1N was incubated with glutathione resin overnight at 4 °C with constant mixing in GST pull-down lysis buffer (20 mM Tris-HCl pH 8.0, 200 mM NaCl, 1 mM EDTA, 0.5% NP-40). Resin was washed 3 times with lysis buffer and incubated with 15 μg of wild-type or mutant MBP-ATG5 for 1 h. Resin was washed 3 times again and proteins were eluted with 30 mM reduced glutathione for 30 min. For GST pull-down assays from wild-type and *ATG7*-knockout PC-3 cell lysates, cells were lysed in ice-cold GST pull-down lysis buffer with protease inhibitors. Lysate concentrations were measured using the Bradford assay, and wild-type and *ATG7*-KO lysates were mixed in a 1:3.5 ratio to equalize the levels of conjugated and unconjugated ATG5. Pull downs were performed as described above with 1.5 mg total lysate. Samples were then evaluated by immunoblotting using antibodies to GST and ATG5.

### FLAG-ubiquitin immunoprecipitation

DU145 cells stably expressing ATG5 ± FLAG-ubiquitin were treated with 10 μM MG132 for 8 h and lysed in ice-cold Triton X-100 lysis buffer (50 mM Tris-HCl, pH 7.4, 150 mM NaCl, 1 mM EDTA and 1% Triton X-100). Lysate concentrations were normalized using the Bradford assay and incubated at 4 °C overnight with 80 μL of ANTI-FLAG M2 Affinity Gel (Sigma-Aldrich #A2220) with constant agitation. Resin was washed three times with ice-cold PBS and boiled in nonreducing Laemmli sample buffer (125 nM Tris-HCl, pH 6.8, 20% glycerol, 4% SDS, 0.1% bromophenol blue). Samples were then evaluated by immunoblotting using antibodies to FLAG and ATG5.

### Bioinformatic analyses

Rendering of the human ATG12 (aa 52–140)–ATG5-ATG16L1N (aa 11–43) (PDB ID: 4GDL) and ATG5-ATG16L1N (PDB ID: 4TQ0) crystal structures, as well as modeling of the ATG16L2N peptide, was performed with the UCSF Chimera package (https://www.cgl.ucsf.edu/chimera/)^27,28,57^. All alternative *ATG5* splice variants and their predicted transcripts were taken from the National Center for Biotechnology Information (NCBI) RefSeq database (https://www.ncbi.nlm.noh.gov/refseq/)^25^. Somatic *ATG5* mutations were collected from the Catalog of Somatic Mutations in Cancer (COSMIC: http://cancer.sanger.ac.uk/cosmic), the International Cancer Genome Consortium (ICGC: https://icgc.org), the Broad Institute Cancer Cell Line Encyclopedia (CCLE: https://portals.broadinstitute.org/ccle), and cBioPortal for Cancer Genomics (http://www.cbioportal.org) databases^58–62^. Somatic mutations from tumor samples were verified with Annotated Somatic Mutation Variant Cell Format (VCF) files associated with each de-identified donor. The effects of *ATG5* splice site mutations on mRNA splicing were predicted, in silico, using the Human Splicing Finder (http://www.umd.be/HSF/)^24^. This algorithm uses position weight matrices to calculate consensus values (CVs), which are the sum of the weighted scores given to each nucleotide based on its position within the conserved splicing motif. The CV variation % refers to the difference in CVs between the wild-type and mutant sequences as a percentage of the wild-type CV.

TCGA level 3 data were downloaded from the TCGA data portal (https://portal.gdc.cancer.gov) and clinical information was extracted. Datasets with <25 normal samples were excluded from the analysis. *ATG5* splice variants *v1*(uc003prf), *v3*(uc003prf.2), *v4*(uc003prg.2), and *v5*(uc010kdb.2) were identified in the TCGA datasets; however, *v4* and *v5* were not expressed in the vast majority of samples. Therefore, the percentage of full-length *ATG5* mRNA (*ATG5v1*; uc003prf) expression was calculated from the sum of *ATG5* (*v1* + *v3*) mRNA expression in normal and tumor tissue and plotted using R. The *ATG16L2*:*ATG16L1* mRNA expression ratio from normal and tumor tissue was also calculated and plotted using R.

### Statistical analyses

All experiments were performed at least three times with each Western blot serving as a representative image. In some experiments, densitometry for bands corresponding to ATG12–ATG5, ATG16L1, and LC3 blots was performed and statistical significance determined using ANOVA with a Student–Newman–Kuels post hoc analysis. For mRNA expression comparisons between tumor and normal tissue from TCGA datasets, unpaired two-tailed *t* tests were performed using R. In all cases *p* < 0.01 was considered statistically significant.

## Data availability

The authors can confirm that all relevant data are included in the paper and/or its Supplementary files.

## Supplementary information

Supplemental Information

Supplemental Table S1

## Supplementary information

**Supplementary Information** accompanies the paper at (10.1038/s41421-019-0110-1).
