# Supplementary material for: ATG5 cancer mutations and alternative mRNA splicing reveal a conjugation switch that regulates ATG12–ATG5-ATG16L1 complex assembly and autophagy
Source: Cell Discov. 2019 Aug 27;5:42. doi: 10.1038/s41421-019-0110-1 (PMC6796855; doi:10.1038/s41421-019-0110-1)
Supplement: Supplementary file 1 — Supplemental Information [file 41421_2019_110_MOESM1_ESM.pdf]

Wible, Fig. S1

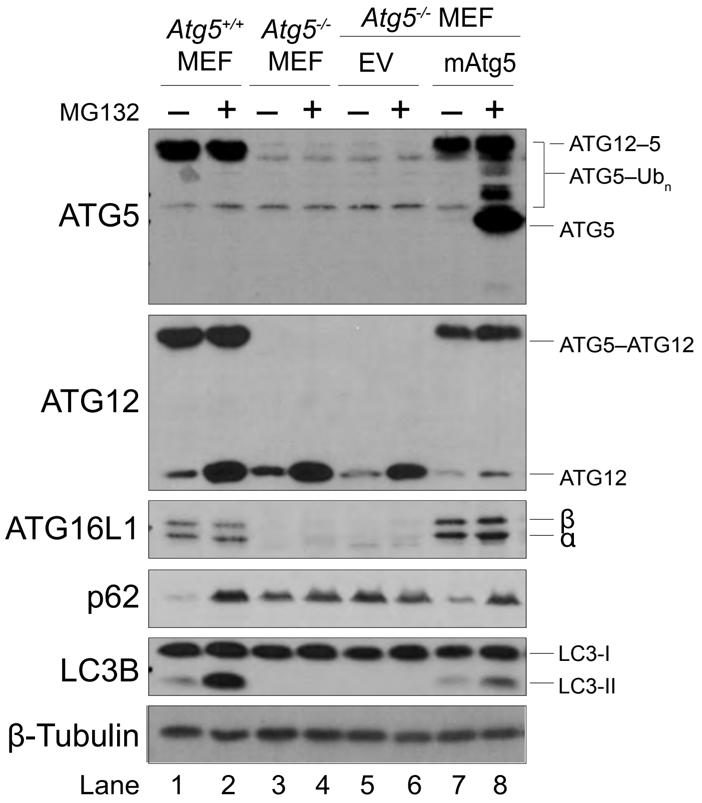

Atg5+/+ MEF
Atg5-/- MEF
Atg5-/- MEF
EV
mAtg5
MG132
– + – + – + – +
ATG5
ATG12–5
ATG5–Ubn
ATG5
ATG12
ATG5–ATG12
ATG12
ATG16L1
β
α
p62
LC3B
LC3-I
LC3-II
β-Tubulin
Lane 1 2 3 4 5 6 7 8

Wible, Fig. S2

a

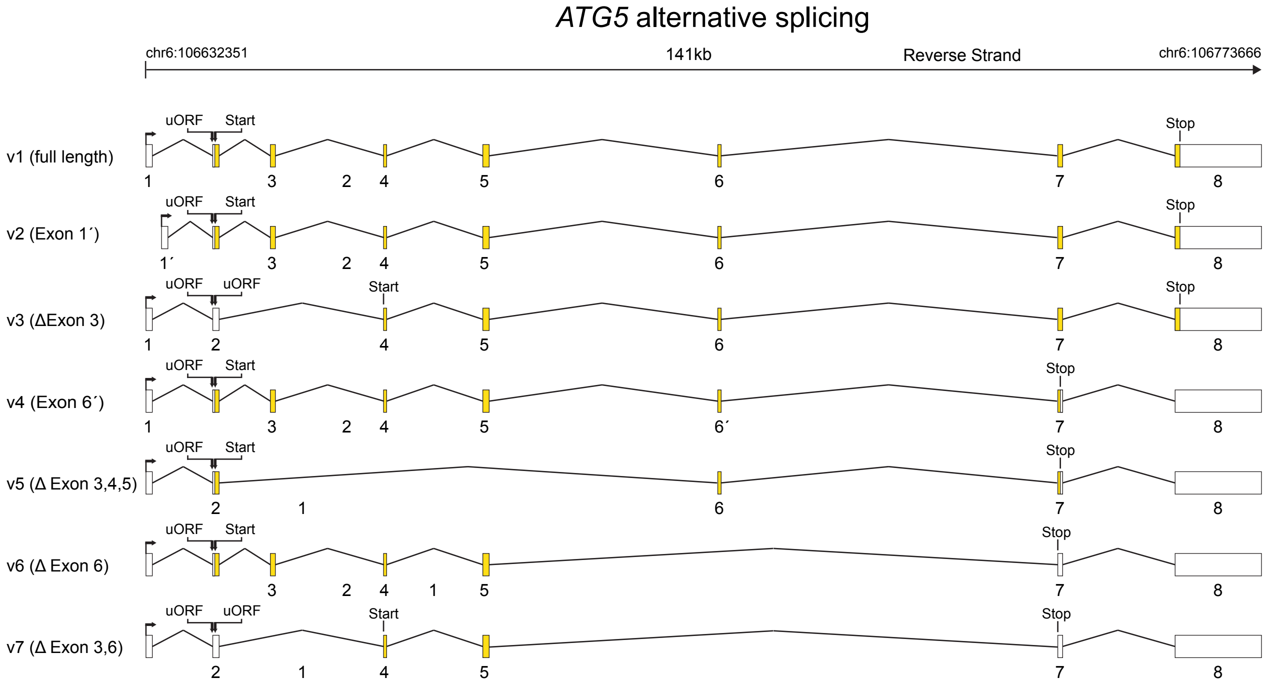


b

| Splice Variant | mRNA Alteration | mRNA Accession Number | Predicted Amino Acid Alteration | Predicted Protein Isoform |
|---|---|---|---|---|
| v1 | Full-length | NM_004849.3 | | a |
| v2 | Exon 1' | NM_001286106.1 | none | a |
| v3 | Δ Exon 3 | NM_001286107.1 | p.M1_W79del | b |
| v4 | Exon 6' | NM_001286108.1 | p.R161Lfs*54 | c |
| v5 | Δ Exon 3,4,5 | NM_001286111.1 | p.L37Tfs*56 | d |
| v6 | Δ Exon 6 | NR_104402.1 | non-coding (p.R161Nfs*3) | (e) |
| v7 | Δ Exon 3,6 | NR_104403.1 | non-coding (p.M1_W79del+p.R162Nfs*3) | (f) |

c

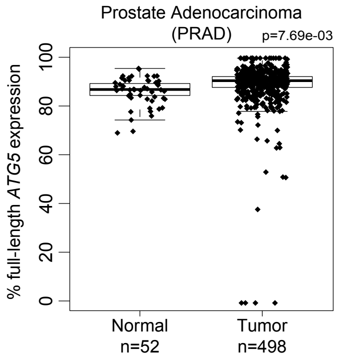

Wible, Fig. S3

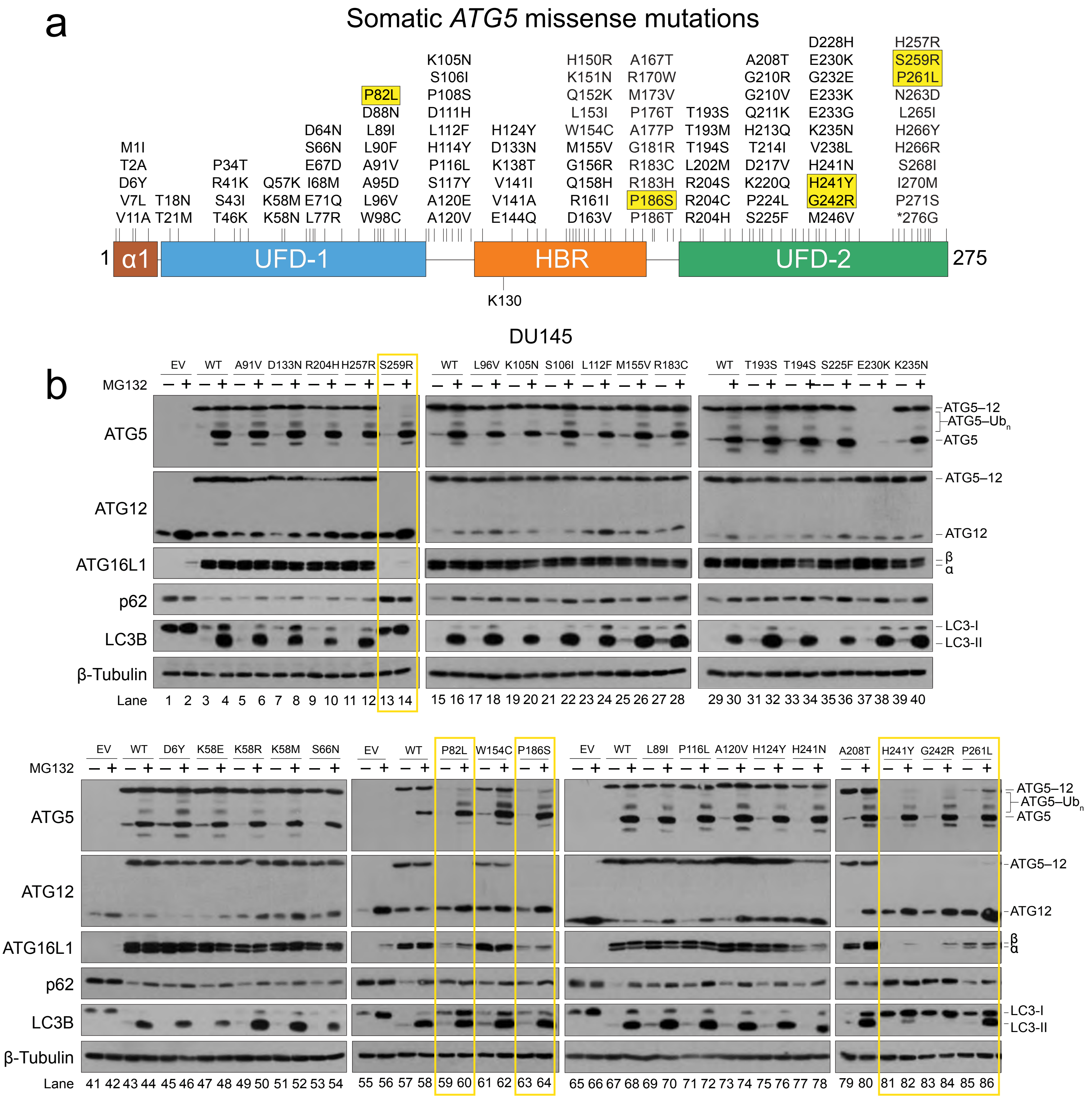
a
Somatic ATG5 missense mutations
M1I
T2A
D6Y
V7L
V11A
T18N
T21M
P34T
R41K
S43I
T46K
Q57K
K58M
K58N
D64N
S66N
E67D
I68M
E71Q
L77R
P82L
D88N
L89I
L90F
A91V
A95D
L96V
W98C
K105N
S106I
P108S
D111H
L112F
H114Y
P116L
S117Y
A120E
A120V
H124Y
D133N
K138T
V141I
V141A
E144Q
H150R
K151N
Q152K
L153I
W154C
M155V
G156R
Q158H
R161I
D163V
A167T
R170W
M173V
P176T
A177P
G181R
R183C
R183H
P186S
P186T
T193S
T193M
T194S
L202M
R204S
R204C
R204H
A208T
G210R
G210V
Q211K
H213Q
T214I
D217V
K220Q
P224L
S225F
D228H
E230K
G232E
E233K
E233G
K235N
V238L
H241N
H241Y
G242R
M246V
H257R
S259R
P261L
N263D
L265I
H266Y
H266R
S268I
I270M
P271S
*276G
1
α1
UFD-1
HBR
UFD-2
275
K130
DU145
b
MG132
EV
WT
A91V
D133N
R204H
H257R
S259R
L96V
K105N
S106I
L112F
M155V
R183C
T193S
T194S
S225F
E230K
K235N
ATG5
ATG12
ATG16L1
p62
LC3B
β-Tubulin
Lane
ATG5–12
ATG5–Ub$_n$
ATG5
ATG12
β
α
LC3-I
LC3-II
1 2 3 4 5 6 7 8 9 10 11 12 13 14
15 16 17 18 19 20 21 22 23 24 25 26 27 28
29 30 31 32 33 34 35 36 37 38 39 40
D6Y
K58E
K58R
K58M
S66N
P82L
W154C
P186S
L89I
P116L
A120V
H124Y
H241N
A208T
H241Y
G242R
P261L
41 42 43 44 45 46 47 48 49 50 51 52 53 54
55 56 57 58 59 60 61 62 63 64
65 66 67 68 69 70 71 72 73 74 75 76 77 78
79 80 81 82 83 84 85 86

Wible, Fig. S4

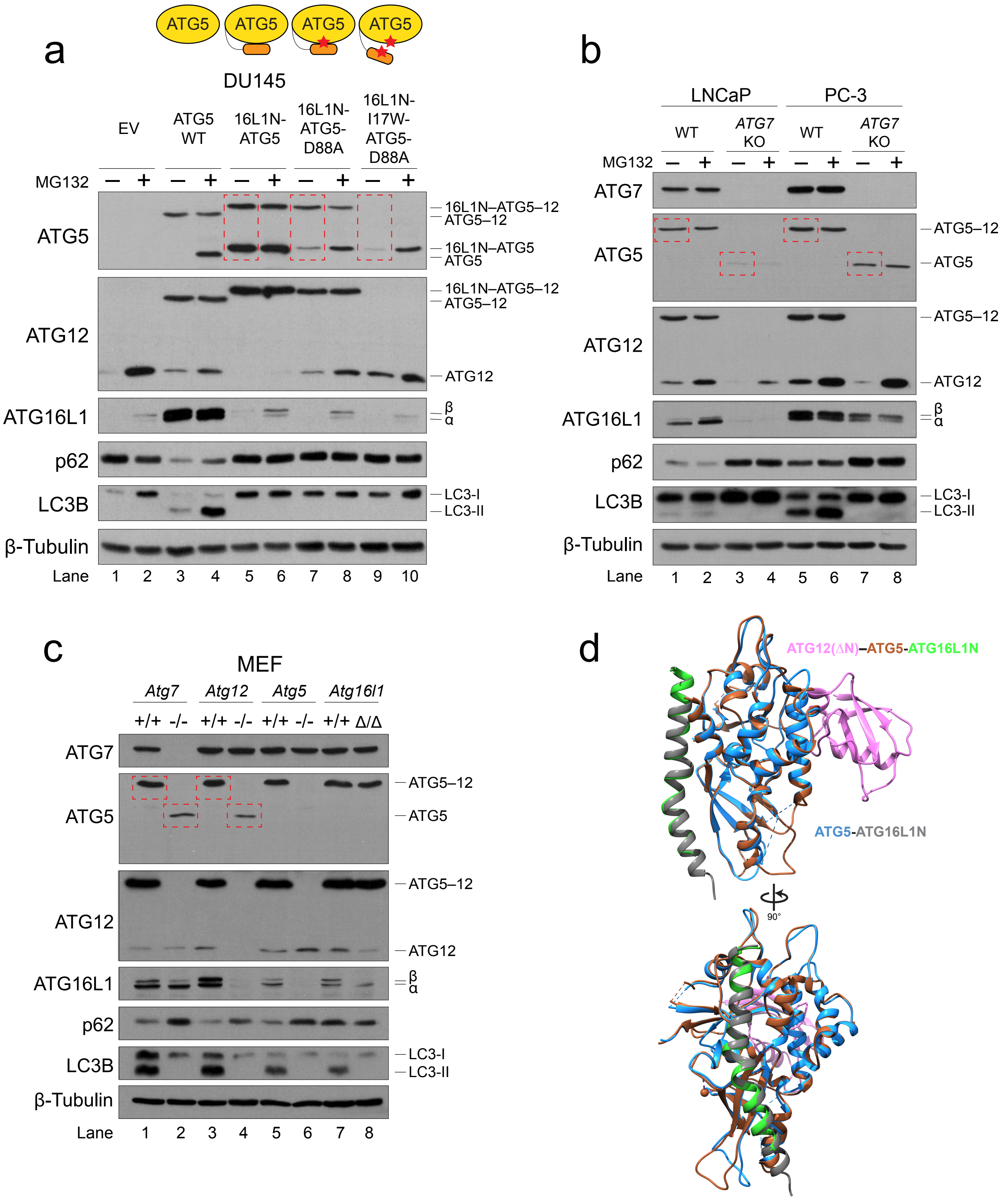
a
ATG5
ATG5
ATG5
ATG5
DU145
EV
ATG5 WT
16L1N-ATG5
16L1N-ATG5-D88A
16L1N-I17W-ATG5-D88A
MG132
ATG5
16L1N–ATG5–12
ATG5–12
16L1N–ATG5
ATG5
ATG12
16L1N–ATG5–12
ATG5–12
ATG12
ATG16L1
β
α
p62
LC3B
LC3-I
LC3-II
β-Tubulin
Lane 1 2 3 4 5 6 7 8 9 10
b
LNCaP
PC-3
WT
ATG7 KO
WT
ATG7 KO
MG132
ATG7
ATG5
ATG5–12
ATG5
ATG12
ATG5–12
ATG12
ATG16L1
β
α
p62
LC3B
LC3-I
LC3-II
β-Tubulin
Lane 1 2 3 4 5 6 7 8
c
MEF
Atg7
Atg12
Atg5
Atg16l1
+/+ -/- +/+ -/- +/+ -/- +/+ Δ/Δ
ATG7
ATG5
ATG5–12
ATG5
ATG12
ATG5–12
ATG12
ATG16L1
β
α
p62
LC3B
LC3-I
LC3-II
β-Tubulin
Lane 1 2 3 4 5 6 7 8
d
ATG12(ΔN)–ATG5-ATG16L1N
ATG5-ATG16L1N
90°

# Wible, Fig. S5

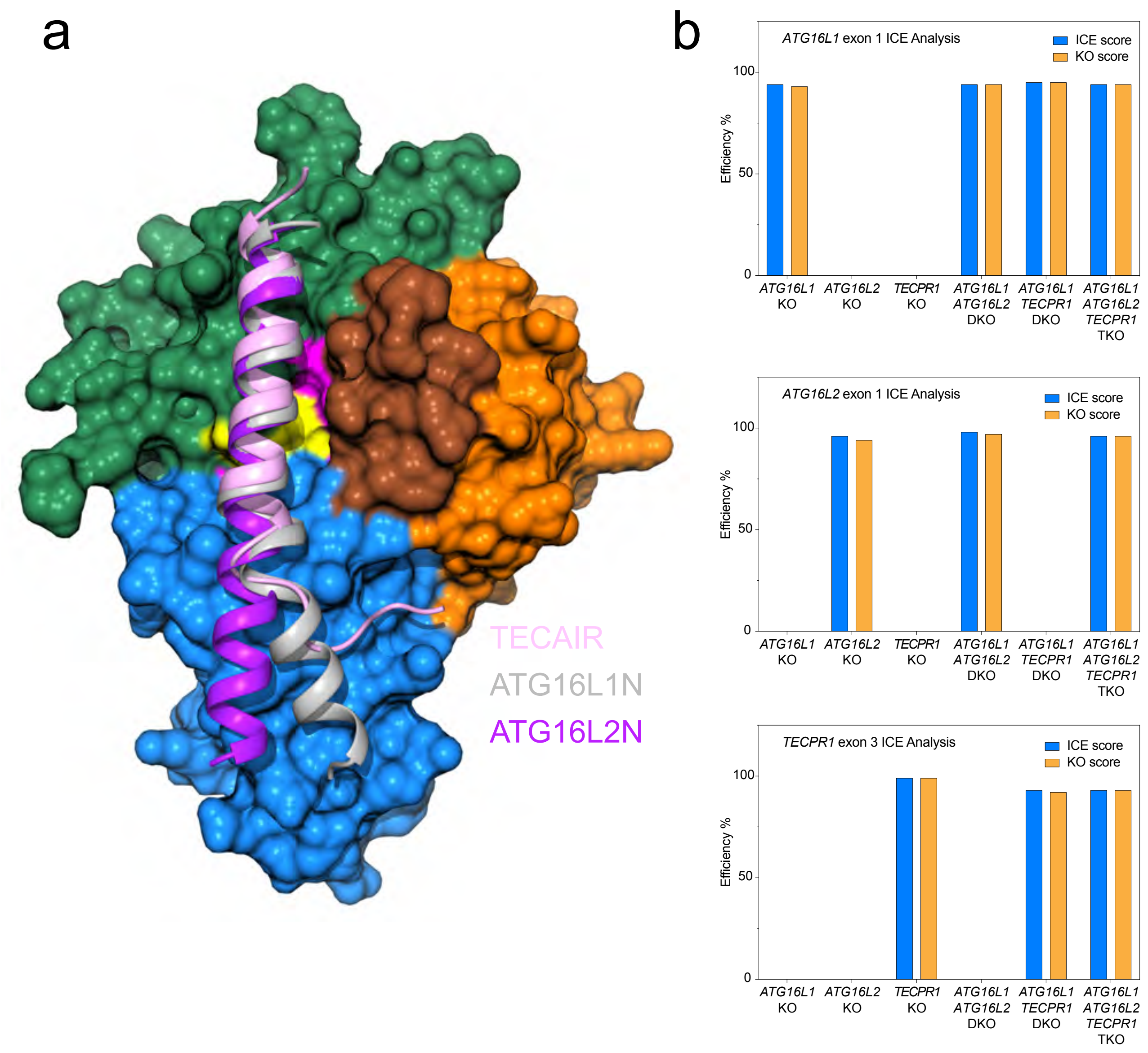

a
TECAIR
ATG16L1N
ATG16L2N
b
ATG16L1 exon 1 ICE Analysis
ICE score
KO score
Efficiency %
100
50
0
ATG16L1 KO
ATG16L2 KO
TECPR1 KO
ATG16L1 ATG16L2 DKO
ATG16L1 TECPR1 DKO
ATG16L1 ATG16L2 TECPR1 TKO
ATG16L2 exon 1 ICE Analysis
TECPR1 exon 3 ICE Analysis

C

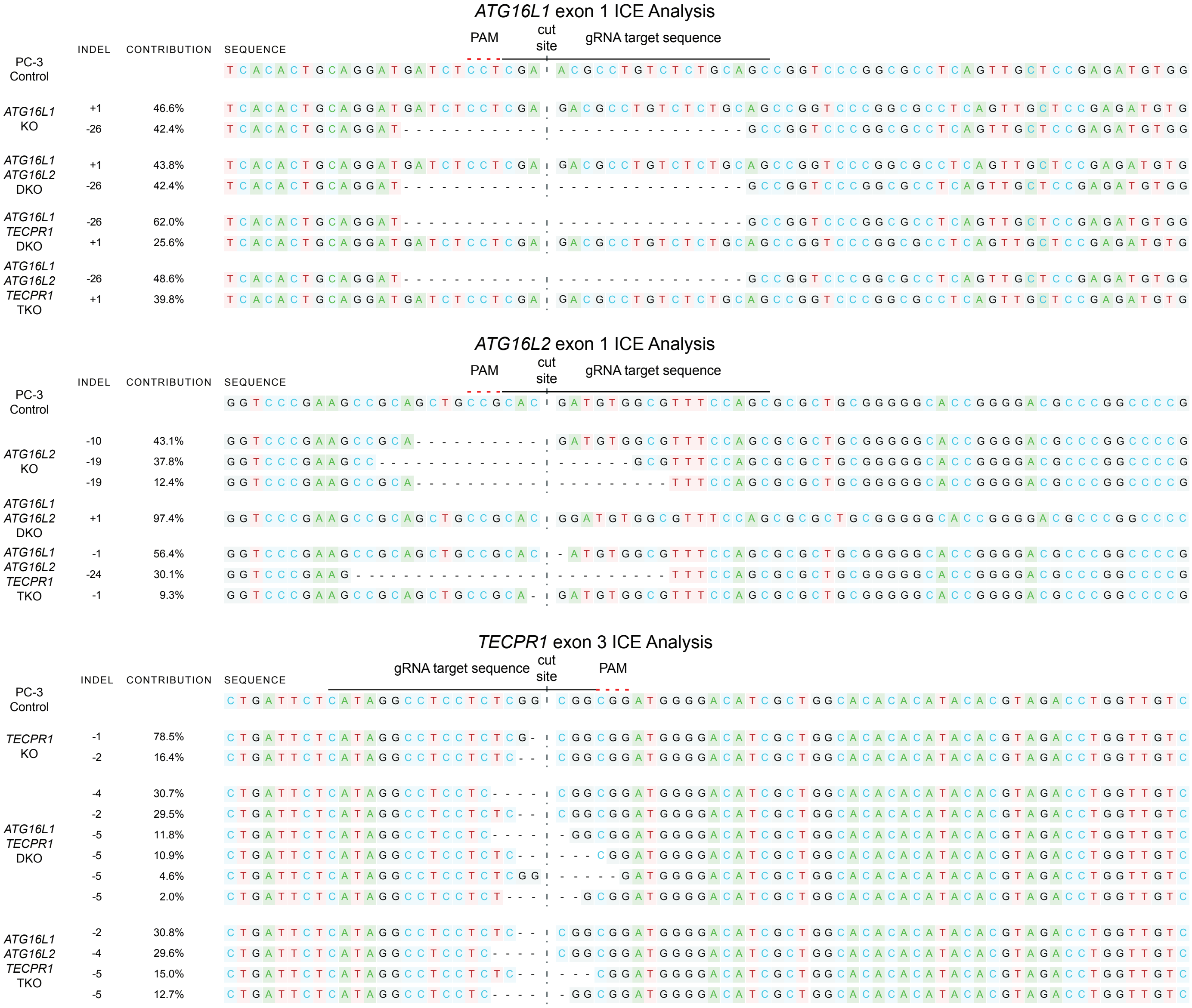

ATG16L1 exon 1 ICE Analysis
INDEL
CONTRIBUTION
SEQUENCE
PAM
cut site
gRNA target sequence
PC-3 Control
ATG16L1 KO
+1
46.6%
-26
42.4%
ATG16L1 ATG16L2 DKO
+1
43.8%
-26
42.4%
ATG16L1 TECPR1 DKO
-26
62.0%
+1
25.6%
ATG16L1 ATG16L2 TECPR1 TKO
-26
48.6%
+1
39.8%
ATG16L2 exon 1 ICE Analysis
INDEL
CONTRIBUTION
SEQUENCE
PAM
cut site
gRNA target sequence
PC-3 Control
ATG16L2 KO
-10
43.1%
-19
37.8%
-19
12.4%
ATG16L1 ATG16L2 DKO
+1
97.4%
ATG16L1 ATG16L2 TECPR1 TKO
-1
56.4%
-24
30.1%
-1
9.3%
TECPR1 exon 3 ICE Analysis
INDEL
CONTRIBUTION
SEQUENCE
gRNA target sequence
cut site
PAM
PC-3 Control
TECPR1 KO
-1
78.5%
-2
16.4%
ATG16L1 TECPR1 DKO
-4
30.7%
-2
29.5%
-5
11.8%
-5
10.9%
-5
4.6%
-5
2.0%
ATG16L1 ATG16L2 TECPR1 TKO
-2
30.8%
-4
29.6%
-5
15.0%
-5
12.7%

## SUPPLEMENTARY FIGURE LEGENDS

**Figure S1. Free ATG5, ATG12 and ATG16L1 undergo protein quality control (PQC) when not incorporated into the ATG12–ATG5-ATG16L1 complex.** *Atg5*$^{+/+}$, *Atg5*$^{-/-}$, and *Atg5*$^{-/-}$ MEFs stably expressing empty vector (EV) or murine *Atg5* (*mAtg5*), were treated with 10 μM MG132 for 8 h and immunoblotted for the indicated proteins.

**Figure S2. Alternative mRNA splicing of *ATG5*.** (**a**) Diagram of known *ATG5* alternative splicing. Variants v6 and v7 were identified in DU145 cells. Cryptic exons are indicated with (′) in variants v2 (Exon 1′) and v4 (Exon 6′). Skipped exons are indicated with (Δ) in variants v3 (ΔExon 3), v5 (ΔExon 3,4,5), v6 (ΔExon 6) and v7 (ΔExon 3,6). The predicted coding regions are indicated in yellow. Upstream open reading frames (uORF) are also indicated. (**b**) Table of known *ATG5* splice variants indicating the mRNA alteration, the NCBI mRNA accession number, and the predicted amino acid alteration. The predicted protein isoforms are designated alphanumerically with the isoforms designated as isoforms 'e' and 'f', predicted to result from variants v6 and v7 in DU145 cells, respectively. (**c**) Tukey boxplot of the *ATG16L2*:*ATG16L1* mRNA expression ratios from normal prostate and tumor samples from the TCGA PRAD dataset.

**Figure S3. Somatic *ATG5* missense mutations disrupt the ATG16L1-binding pocket.** **(a)** A diagram of all *ATG5* missense mutations identified in human tumors and their locations within the ATG5 domain structure is shown above. (**b**) DU145

cells, stably expressing empty vector (EV), wild-type ATG5 (WT), or one of the missense mutants, were treated with 10 μM MG132 for 8 h and immunoblotted for the indicated proteins. Missense mutations that dramatically impaired ATG12 conjugation and LC3B conjugation are highlighted in yellow. ATG5-E230K was not detected with the ATG5 antibody because the E230K mutation disrupted the ATG5 antibody epitope; however, ATG12–ATG5 conjugate was still detected by the ATG12 antibody (lane 37-38).

**Figure S4. ATG12 conjugation to ATG5 stabilizes a transient ATG5-ATG16L1 interaction.** (**a**) DU145 cells, stably expressing empty vector (EV), wild-type ATG5, wild-type ATG5 tethered to an N-terminal fragment of ATG16L1 (16L1N-ATG5), or internal binding mutants of 16L1N-ATG5, were treated with 10 μM MG132 for 8 h and immunoblotted for the indicated proteins. A cartoon depicting each of the different expression constructs is shown above. Notably, 16L1N-ATG5 is fully stable and does not undergo proteasomal degradation (lanes 5-6). Introduction of the D88A mutation into ATG5 partially disrupts the 16L1N-linker from binding, but still allows some inefficient ATG12 conjugation (lanes 7-8). Mutation of both the 16L1N linker (I17W) and ATG5 (D88A), however, resulted in complete degradation of the fusion protein (lanes 9-10). (**b**) LNCaP and PC-3 *ATG7* CRISPR/Cas9 knockout cell lines (*ATG7* KO) were treated with 10 μM MG132 for 8 h and immunoblotted for the indicated proteins. (**c**) Lysates from *Atg7*, *Atg12*, and *Atg5* wild-type (+/+) and knockout (-/-) MEFs were immunoblotted for the indicated proteins. Additionally, lysates from

wild-type MEFs and those expressing a form of *Atg16l1* that lacks its coiled-coil domain (Δ/Δ) were immunoblotted for the indicated proteins (lane 7-8). (**d**) Crystal structures for ATG12ΔN–ATG5-ATG16L1N (PDB ID: 4GDL) and ATG5-ATG16L1N (PDB ID: 4TQ0) were overlaid.

**Figure S5. ICE analysis of *ATG16L1*$^{-/-}$, *ATG16L2*$^{-/-}$, and *TECPR1*$^{-/-}$ PC-3 prostate cancer cells.** **(a)** Crystal structures of ATG16L1N (PDB: 4TQ0) and TECAIR (PDB: 4TQ1) bound to ATG5, and a model for ATG16L2N bound to the same pocket. **(b)** Inference of CRISPR Edits (ICE) Analysis of *ATG16L1* exon 1, *ATG16L2* exon 1 and *TECPR1* exon 3 in *ATG16L1* KO, *ATG16L2* KO, *TECPR1* KO, *ATG16L1*/*L2* DKO, *ATG16L1*/*TECPR1* DKO and *ATG16L1*/*L2*/*TECPR1* TKO PC-3 cells. ICE score refers to the percentage of cells possessing an insertion or deletion (indel). KO score refers to the percentage of cells possessing an indel predicted to cause a frameshift and a loss of gene function. (**c**) Sequence alignment of predicted indels in *ATG16L1*, *ATG16L2* and *TECPR1* from the ICE analysis.

**Table S1. Somatic nonsynonymous *ATG5* coding sequence mutations in human tumor samples and cancer cell lines.** Somatic mutations identified in human tumor samples and cancer cell lines were divided into nonsense, deletion, insertion and missense categories. The DNA alteration (CDS) and predicted amino acid alteration for each mutation are listed along with the tumor type and sequencing ID from which the mutation was identified. The tumor sample count indicates the number of unique

tumors or cancer cell lines in which specific mutation was found. Select mutants were stably expressed in DU145 cells and evaluated for their ability to prevent ATG16L1 degradation and promote ATG12 conjugation, LC3B lipidation and p62 degradation. Mutations causing complete or partial loss of function are indicated in bold. Mutations that were confirmed to have no effect are labeled with ‘No’, while mutations that were not tested are left blank.

**Supplementary Table 2. RT-PCR primer sequences**

| Gene | Sequence (5′-3′) |
|---|---|
| *ATG5* (human) | F- GTG ACG TCA TCT CCG GGC GC<br>R- TGA AGC AAA AGG GTG ACA TGC TC |

**Supplementary Table 3. Genomic PCR primer sequences**

| Gene | Sequence (5′-3′) |
|---|---|
| *ATG5* exon 6 (human) | F- CAG AAA CTT CTA GAG GGA TAT TTA<br>R- ACC GTT TAG TTA CTA TGC AGA CAA |
| *ATG16L1* exon 1 (human) | F- TCT TCC GGC CCT CTC GAA<br>R- CGA CTG AAA GTC AAC GAC CG |
| *ATG16L2* exon 1 (human) | F- GAG CCC CCA CTT AGG TGA AC<br>R- CTC AAG GCA TAA GGC ACA GC |
| *TECPR1* exon 3 (human) | F- CCA TGC CCA ACT CAG TGC T<br>R- ATG AAG GCA GGA ACT GGT GG |

**Supplementary Table 4. sgRNA oligo sequences**

| Gene | Sequence (5′-3′) |
|---|---|
| *ATG5* (human) | F- cac cgA TCA GGA TGA GAT AAC TGA A<br>R- aaa cTT CAG TTA TCT CAT CCT GAT c |
| *ATG5* exon 6 (human) | F- cac cgC CCT TTA GAA TAT ATC AGG T<br>R- aaa cAC CTG ATA TAT TCT AAA GGG c |
| *ATG7* (human) | F- cac cgG CGG CAG CTA CGG GGG ATC C<br>R- aaa cGG ATC CCC CGT AGC TGC CGC c |
| *ATG16L1* (human) | F- cac cgG CTG CAG AGA CAG GCG TTC G<br>R- aaa cCG AAC GCC TGT CTC TGC AGC c |
| *ATG16L2* (human) | F- cac cgG CTG GAA ACG CCA CAT CGT G<br>R-aaa cCA CGA TGT GGC GTT TCC AGC C |
| *TECPR1* (human) | F- cac cgC ATA GGC CTC CTC TCG GCG G<br>R- aaa cCC GCC GAG AGG AGG CCT ATG C |

**Supplementary Table 5. ssODN donor template sequence**

| Gene | Sequence (5′-3′) |
|---|---|
| *ATG5 c.573+1A>G* | 5′- AGT GCT TCA ATT AAG ATG TCT GAG GCT TTC ATA AAT GGA TGT TTT TTA AAA TGT TAT TTC CTA cCT GgT AaA TaC gAA AGG GGA TAT AAC GAA ATC CAT TTT CTT CTG CAG GAT ATT CCA TGA GTT TCC G - 3′ |

**Supplementary Table 6. Cloning primer sequences**

| Gene | Sequence (5′-3′) |
|---|---|
| *ATG5* (v1; full-length; isoform a) | F- GGA GGA TCC ATG ACA GAT GAC AAA GAT GTG C<br>R (stop codon)- GCT GCT AGC TCA ATC TGT TGG CTG TGG GAT G<br>R (no stop codon)- GCT GCT AGC ATC TGT TGG CTG TGG GAT GAT A |
| *ATG5* (v3; ΔExon3; isoform b) | F- GGA GGA TCC ATG CAT TAT CCA ATT GGT TTG CTA T<br>R- same as *ATG5*v1 |
| *ATG5* (v4; Exon6′; isoform c) | F- same as *ATG5*v1<br>R- GCT GCT AGC GTG TGT GCA ACT GTC CAT CTG |
| *ATG5* (v5; ΔExon3,4,5; isoform d) | F- same as *ATG5*v1<br>R- GCT GCT AGC ATC TGT TGG CTG TGG GAT GAT AC |
| *ATG5* (v6; ΔExon6; isoform e) | F- same as *ATG5*v1<br>R- GCT GCT AGC GTG TGT GCA ACT GTC CAT CTG |
| *ATG5* (v7; ΔExon3,6; isoform f) | F- same as *ATG5*v3<br>R- same as *ATG5*v6 |
| ATG5 p.M1_V11del (c.28_31delGATG) | F- GGA GAA TCC ATG TGG TTT GGA CGA ATT CCA AC<br>R- same as *ATG5*v1 |
| ATG5 p.M1_V59del (c.3G>A; c.25C>T; c.43C>T; c.41delG) | F- GGA GAA TCC ATG AGA CAA GAA GAC ATT AGT GAG<br>R- same as *ATG5*v1 |
| GS-linker-*ATG5* | F- GCT GCT AGC GGT GGA GGA GGT TCT ACA GAT GAC AAA GAT GTG CTT G<br>R- ATA GCG CGC CTC AAT CTG TTG GCT GTG GGA TG |
| *ATG5* (mouse) | F- same as *ATG5*v1<br>R- GCT GCT AGC TCA ATC TGT TGG CTG GGG GAC AAT G |
| *ATG16L1* | F- GGA GGA TCC ATG TCG TCG GGC CTC CGC G<br>R- GCT GCT AGC GTA CTG TGC CCA CAG CAC AGC |
| *ATG16L1ΔN* (nucleotides 118-1,824) | F- GGA GGA TCC ATG AAC AAA TTG CTG GAA AAG TCA GAT C<br>R- same as *ATG16L1* |
| *ATG16L1N* (nucleotides 1-207) | F- same as *ATG16L1*<br>R- same as *AAT GCG GCC GCT CAT ATC TCG TGC CTG TTT GGT A* |
| *ATG16L1N* (nucleotides 31-108) | F- GGA GGA TCC ATG CCC CGC TGG AAG CGC CAC<br>R- GCT GCT AGC GAT GAT CTC CTC GAA CGC CTG |

| | |
|---|---|
| *ATG16L2* | F- GGA GGA TCC ATG GCG GGG CCG GGC GTC<br>R- GCT GCT AGC CTG CCA GAG CAC AAC CTT CC |
| *ATG16L2ΔN* (nucleotides 109-1,860) | F- GGA GGA TCC ATG TAT AAC CAT CTC TTA GAG AAG GC<br>R- same as *ATG16L2* |
| *ATG16L2N* (nucleotides 1-207) | F- same as *ATG16L2*<br>R- AAT GCG GCC GCT CAG TGG GTG GTG GGA GTG AC |
| *TECPR1* | F- GCT GCT AGC CCC AAC TCA GTG CTG TGG GC<br>R- GAA GGC GCG CCT CAG CAG CAG ACG GGG CCA T |
| *TECPR1* TECAIR (nucleotides 1,696-1,830) | F- GCT GCT AGC CCC AAC TCA GTG CTG TGG GC<br>R- GAA GGC GCG CCT CAG CAG CAG ACG GGG CCA T |
| *Ubiquitin* | F- GCT GCT AGC CAG ATT TTC GTG AAA ACC CTT ACG<br>R- GAA GGC GCG CCT TTA ACC ACC ACG AAG TCT CAA CA |

**Supplementary Table 7. Site-directed mutagenesis primer sequences**

| **Gene** | **Sequence (5′-3′)** |
|---|---|
| **Structure-directed *ATG5* mutations** | |
| ATG5 V7S (c.19_20GT>TC) | F- GAC AGA TGA CAA AGA TTC GCT TCG AGA TGT GTG G<br>R- CCA CAC ATC TCG AAG CGA ATC TTT GTC ATC TGT C |
| ATG5 D10A (c.29A>T) | F- AAG ATG TGC TTC GAG CTG TGT GGT TTG GAC G<br>R- CGT CCA AAC CAC ACA GCT CGA AGC ACA TCT T |
| ATG5 V11S (c.31_32GT>TC) | F- AGA TGT GCT TCG AGA TTC GTG GTT TGG ACG AAT T<br>R- AAT TCG TCC AAA CCA CGA ATC TCG AAG CAC ATC T |
| ATG5 G14S (c.40G>T) | F- TCG AGA TGT GTG GTT TTC ACG AAT TCC AAC TTG T<br>R- ACA AGT TGG AAT TCG TGA AAA CCA CAC ATC TCG A |
| ATG5 R41A (c.121_122AG>GC) | F- ACT ATT TGC TTT TGC CAG CAG TAA GTT ATT TGA CGT<br>R- ACG TCA AAT AAC TTA CTG CTG GCA AAA GCA AAT AGT |
| ATG5 G84S (c.250G>A) | F- AAA TGG CAT TAT CCA ATT AGT TTG CTA TTT GAT CTT C<br>R- GAA GAT CAA ATA GCA AAC TAA TTG GAT AAT GCC ATT T |
| ATG5 D88A (c.263A>C) | F- AAT TGG TTT GCT ATT TGC TCT TCT TGC ATC AAG TTC<br>R- GAA CTT GAT GCA AGA AGA GCA AAT AGC AAA CCA ATT |
| ATG5 I240S (c.719T>G) | F- AAA GAA TCA AGT GAT GAG TCA TGG AAT TGA GCC AA<br>R- TTG GCT CAA TTC CAT GAC TCA TCA CTT GAT TCT TT |

| | |
|---|---|
| ATG5 I243S (c.728T>G) | F- GTG ATG ATT CAT GGA AGT GAG CCA ATG TTG GAA<br>R- TTC CAA CAT TGG CTC ACT TCC ATG AAT CAT CAC |
| ATG5 L254S (c.760_761CT>TC) | F- ACA CCT CTG CAG TGG TCG AGT GAA CAT CTG AGC<br>R- GCT CAG ATG TTC ACT CGA CCA CTG CAG AGG TGT |
| ATG5 L258S (c.772_773CT>TC) | F- GTG GCT GAG TGA ACA TTC GAG CTA CCC GGA TAA<br>R- TTA TCC GGG TAG CTC GAA TGT TCA CTC AGC CAC |
| **Somatic *ATG5* nonsense and deletion mutations** | |
| ATG5 p.W12_F12delinsC (c.36_38delGTT) | F- GCT TCG AGA TGT GTG TGG ACG AAT TCC AAC<br>R- GTT GGA ATT CGT CCA CAC ACA TCT CGA AGC |
| ATG5 E144* (c.430G>T) | F- AAA AGT CAA GTA ATC AAT TAA ATG CAG AAA AAA GAT C<br>R- GAT CTT TTT TCT GCA TTT AAT TGA TTA CTT GAC TTT T |
| ATG5 E179* (c.535G>T) | F- TGG AAT ATC CTG CAG AAT AAA ATG GAT TTC GTT AT<br>R- ATA ACG AAA TCC ATT TTA TTC TGC AGG ATA TTC CA |
| ATG5 p.K235Rfs*4 (c.704delA) | F- CTG AAG ATG GGG AAA AAA GAA TCA AGT GAT GAT TC<br>R- GAA TCA TCA CTT GAT TCT TTT TTC CCC ATC TTC AG |
| **Somatic *ATG5* missense mutations** | |
| ATG5 K58M (c.173A>T) | F- GAA AAA GCA CTT TCA GAT GGT TAT GAG ACA AGA AG<br>R- CTT CTT GTC TCA TAA CCA TCT GAA AGT GCT TTT TC |
| ATG5 S66N (c.197G>A) | F- GAG ACA AGA AGA CAT TAA TGA GAT ATG GTT TGA AT<br>R- ATT CAA ACC ATA TCT CAT TAA TGT CTT CTT GTC TC |
| ATG5 P82L (c.245C>T) | F- CAC TGA AAT GGC ATT ATC TAA TTG GTT TGC TAT TTG<br>R- CAA ATA GCA AAC CAA TTA GAT AAT GCC ATT TCA GTG |
| ATG5 L89I (c.265C>A) | F- TTG GTT TGC TAT TTG ATA TTC TTG CAT CAA GTT CA<br>R- TGA ACT TGA TGC AAG AAT ATC AAA TAG CAA ACC AA |
| ATG5 A91V (c.272C>T) | F- GCT ATT TGA TCT TCT TGT ATC AAG TTC AGC TCT TC<br>R- GAA GAG CTG AAC TTG ATA CAA GAA GAT CAA ATA GC |
| ATG5 L96V (c.286C>G) | F- GCA TCA AGT TCA GCT GTT CCT TGG AAC ATC AC<br>R- GTG ATG TTC CAA GGA ACA GCT GAA CTT GAT GC |
| ATG5 K105N (c.315G>T) | F- ATC ACA GTA CAT TTT AAT AGT TTT CCA GAA AAA GAC<br>R- GTC TTT TTC TGG AAA ACT ATT AAA ATG TAC TGT GAT |
| ATG5 S106I (c.317G>T) | F- CAC AGT ACA TTT TAA GAT TTT TCC AGA AAA AGA CC<br>R- GGT CTT TTT CTG GAA AAA TCT TAA AAT GTA CTG TG |
| ATG5 L112F (c.334C>T) | F- GTT TTC CAG AAA AAG ACT TTC TGC ACT GTC CAT C<br>R- GAT GGA CAG TGC AGA AAG TCT TTT TCT GGA AAA C |

| | |
|---|---|
| ATG5 P116L (c.346_347CC>TT) | F- AGA CCT TCT GCA CTG TTT ATC TAA GGA TGC AAT T<br>R- AAT TGC ATC CTT AGA TAA ACA GTG CAG AAG GTC T |
| ATG5 A120V (c.359C>T) | F- CTG TCC ATC TAA GGA TGT AAT TGA AGC TCA TTT TA<br>R- TAA AAT GAG CTT CAA TTA CAT CCT TAG ATG GAC AG |
| ATG5 H124Y (c.370C>T) | F- AGG ATG CAA TTG AAG CTT ATT TTA TGT CAT GTA TG<br>R- CAT ACA TGA CAT AAA ATA AGC TTC AAT TGC ATC CT |
| ATG5 D133N (c.397G>A) | F- TCA TGT ATG AAA GAA GCT AAT GCT TTA AAA CAT AAA A<br>R- TTT TAT GTT TTA AAG CAT TAG CTT CTT TCA TAC ATG A |
| ATG5 M155V (c.463A>G) | F- TCA CAA GCA ACT CTG GGT GGG ATT GCA AAA TG<br>R- CAT TTT GCA ATC CCA CCC AGA GTT GCT TGT GA |
| ATG5 R183C (c.547C>T) | F- CAG AAG AAA ATG GAT TTT GTT ATA TCC CCT TTA GA<br>R- TCT AAA GGG GAT ATA ACA AAA TCC ATT TTC TTC TG |
| ATG5 P186S (c.556C>T) | F- AAT GGA TTT CGT TAT ATC TCC TTT AGA ATA TAT CAG AC<br>R- GTC TGA TAT ATT CTA AAG GAG ATA TAA CGA AAT CCA TT |
| ATG5 T193S (c.577A>T) | F- TTA GAA TAT ATC AGA CAT CGA CTG AAA GAC CTT TC<br>R- GAA AGG TCT TTC AGT CGA TGT CTG ATA TAT TCT AA |
| ATG5 T194S (c.580A>T) | F- GAA TAT ATC AGA CAA CGT CTG AAA GAC CTT TCA TTC<br>R- GAA TGA AAG GTC TTT CAG ACG TTG TCT GAT ATA TTC |
| ATG5 R204H (c.611G>A) | F- ATT CAG AAG CTG TTT CAT CCT GTG GCT GCA GAT<br>R- ATC TGC AGC CAC AGG ATG AAA CAG CTT CTG AAT |
| ATG5 A208T (c.622G>A) | F- TTT CGT CCT GTG GCT ACA GAT GGA CAG TTG C<br>R- GCA ACT GTC CAT CTG TAG CCA CAG GAC GAA A |
| ATG5 S225F (c.674C>T) | F- CAA AGA AGT TTG TCC TTT TGC TAT TGA TCC TGA A<br>R- TTC AGG ATC AAT AGC AAA AGG ACA AAC TTC TTT G |
| ATG5 E230K (c.688G>A) | F- TTC TGC TAT TGA TCC TAA AGA TGG GGA AAA AAA G<br>R- CTT TTT TTC CCC ATC TTT AGG ATC AAT AGC AGA A |
| ATG5 K235N (c.705G>T) | F- GAA GAT GGG GAA AAA AAC AAT CAA GTG ATG ATT C<br>R- GAA TCA TCA CTT GAT TGT TTT TTT CCC CAT CTT C |
| ATG5 H241N (c.721C>A) | F- AGA ATC AAG TGA TGA TTA ATG GAA TTG AGC CAA TG<br>R- CAT TGG CTC AAT TCC ATT AAT CAT CAC TTG ATT CT |
| ATG5 H241Y (c.721C>T) | F- GAA TCA AGT GAT GAT TTA TGG AAT TGA GCC AAT G<br>R- CAT TGG CTC AAT TCC ATA AAT CAT CAC TTG ATT C |
| ATG5 G242R (c.724G>A) | F- ATC AAG TGA TGA TTC ATA GAA TTG AGC CAA TGT TG<br>R- CAA CAT TGG CTC AAT TCT ATG AAT CAT CAC TTG AT |

| | |
|---|---|
| ATG5 H257R (c.770A>G) | F- AGT GGC TGA GTG AAC GTC TGA GCT ACC CGG<br>R- CCG GGT AGC TCA GAC GTT CAC TCA GCC ACT |
| ATG5 S259R (c.775A>C) | F- GCT GAG TGA ACA TCT GCG CTA CCC GGA TAA TTT<br>R- AAA TTA TCC GGG TAG CGC AGA TGT TCA CTC AGC |
| ATG5 P261L (c.782C>T) | F- GTG AAC ATC TGA GCT ACC TGG ATA ATT TTC TTC ATA<br>R- TAT GAA GAA AAT TAT CCA GGT AGC TCA GAT GTT CAC |
| **Non-conjugatable *ATG5* mutation** | |
| ATG5 K130R (c.389A>G) | F- TTT TAT GTC ATG TAT GAG AGA AGC TGA TGC TTT AA<br>R- TTA AAG CAT CAG CTT CTC TCA TAC ATG ACA TAA AA |
| ***ATG16L1 Bam*HI-knockout mutation** | |
| *ATG16L1* (c.1347A>G) | F- GCT GGA AGC GCC ACT GGT CGG AGC AAC TGA G<br>R- CTC AGT TGC TCC GAC CAG TGG CGC TTC CAG C |
| ***ATG16L1* ATG5-binding mutation** | |
| ATG16L1 I17W (c.49_51ATC>TGG) | F- AAG ACA GTG TTT GCA GGG TCC AGT TGC AAT GAT A<br>R- TAT CAT TGC AAC TGG ACC CTG CAA ACA CTG TCT T |
| ***ATG16L2* ATG5-binding mutation** | |
| ATG16L2 I18W (c.52_54ATC>TGG) | F- GCT GGA AAC GCC ACT GGG TGC GGC AGC TGC G<br>R- CGC AGC TGC CGC ACC CAG TGG CGT TTC CAG C |
| ***TECPR1* ATG5-binding mutation** | |
| TECPR1 I582W (c.1744_1746ATC>TGG) | F- GCC TGG AGG AAG CAG TGG TTC CAG CAG CTC AC<br>R- GTG AGC TGC TGG AAC CAC TGC TTC CTC CAG GC |
