# Supplementary material for: ATG5 cancer mutations and alternative mRNA splicing reveal a conjugation switch that regulates ATG12–ATG5-ATG16L1 complex assembly and autophagy
Source: Cell Discov. 2019 Aug 27;5:42. doi: 10.1038/s41421-019-0110-1 (PMC6796855; doi:10.1038/s41421-019-0110-1)
Supplement: Supplementary file 2 — Supplemental Table S1 [file 41421_2019_110_MOESM2_ESM.pdf]

Wible Table S1

Somatic Nonsynonymous *ATG5* Coding Sequence Mutations in Human Tumor Samples and Cancer Cell Lines

Mutation Type	Mutation (CDS)	Predicted Amino Acid Alteration	Tumor type	Sequencing ID	Tumor Sample Count	Confirmed Loss of Function (this study)
Nonsense						
	**c.25C>T**	**p.R9* OR p.M1_V59del**	**Glioblastoma multiforme; Lower grade glioma**	**TCGA-06-2562; TCGA-DU-6404**	**2**	**Complete**
	**c.43C>T**	**p.R15* OR p.M1_V59del**	**Prostate adenocarcinoma; Lower grade glioma; Hepatocellular carcinoma; Colorectal adenocarcinoma**	**TCGA-EJ-5512; TCGA-CS-5390; HCC070 (ICGC - China); T2448 (Giannakis et al., *Cell Rep*, 2016)**	**4**	**Complete**
	c.157A>T	p.K53*	Hepatocellular carcinoma	BCM275 (ICGC - France)	1	
	c.169C>T	p.Q57*	Squamous cell carcinoma	CSCC-4 (Pickering et al., *Clin Cancer Res*, 2014)	1	
	**c.430G>T**	**p.E144***	**Colorectal adenocarcinoma**	**TCGA-AG-A002**	**1**	**Complete**
	c.454C>T	p.Q152*	Uterine corpus endometrial carcinoma	TCGA-AX-A1CE	1	
	**c.535G>T**	**p.E179***	**Colorectal adenocarcinoma**	**587376 (Seshagiri et al., *Nature*, 2012)**	**1**	**Complete**
Deletion						
	c.26_28delGAG	p.R9_D10delinsH	Hepatocellular carcinoma	TCGA-5R-AA1C	1	
	**c.28_31delGATG**	**p.M1_V11del**	**Hepatocellular carcinoma**	**HX19 (ICGC - Japan)**	**1**	**Complete**
	**c.36_38delGTT**	**p.W12_F13delinsC**	**Hepatocellular carcinoma**	**BN42 (ICGC - Japan)**	**1**	**Complete**
	c.41delG	p.G14fs*14 OR p.M1_V59del	Cutaneous melanoma	Pat_63_A (Van Allen et al., *Cancer Discov.*, 2014)	1	
	**c.704delA**	**p.K235Rfs*4**	**Prostate adenocarcinoma metastasis; Ampullary carcinoma; Biliary tract cancer; Colorectal adenocarcinoma; Gastric adenocarcinoma; Ovarian adenocarcinoma; Uterine corpus endometrial carcinoma**	**LNCaP clone FGC cell line (CCLE - LNCAPCLONEFGC_PROSTATE); TUD-DA-781 (Gingras et al., *Cell Rep*, 2016); BD124 (ICGC - Japan); TCGA-A6-2672; TCGA-D5-6930; TCGA-A6-2686; NCI_H630 cell line (CCLE - NCIH630_LARGE_INTESTINE); SNU-1040 cell line (CCLE - SNU1040_LARGE_INTESTINE); Kang et al., *J. Pathology*, 2009; TCGA-CD-A4MJ, TCGA-BR-4368; TCGA-BR-4292; TCGA-F1-A448; IM95 cell line (CCLE - IM95_STOMACH); EFO-27 cell line (CCLE - EFO27_OVARY); TCGA-D1-A16F; TCGA-B5-A3FC**	**17**	**Complete**
Insertion						
	c.704dupA	p.E236Efs*9	Colorectal adenocarcinoma	TCGA-AA-3949	1	
	c.704A>AG	p.E236Nfs*9	Cholangiocarcinoma metastasis; Sporadic Burkitt's lymphoma	MO_1347 (Robinson et al., *Nature*, 2017); sBL_01 (Kaymaz et al., *Mol Cancer Biol.*, 2017)	2	
	c.740_741insCAACCATGGTCACCTTA	p.L247Ffs*14	Uterine corpus endometrial carcinoma	TCGA-BS-A0UT	1	
	c.787_788insCTTCC	p.N263Tfs*48	Uterine corpus endometrial carcinoma	TCGA-BS-A0UJ	1	
Missense						
	**c.3G>A**	**p.M1I (p.M1_V59del)**	**Hepatocellular carcinoma**	**CHG-13-08463 (ICGC - China)**	**1**	**Complete**
	c.4A>G	p.T2A	Uterine corpus endometrial carcinoma	TCGA-E6-A1LX	1	
	c.16G>T	p.D6Y	Colorectal adenocarcinoma; Uterine endometriod carcinoma	TCGA-AA-3977; TCGA-AJ-A5DW	2	No
	c.19G>T	p.V7L	Hepatocellular carcinoma	HCC111 (ICGC - China)	1	
	c.32T>C	p.V11A	Colorectal adenocarcinoma	TCGA-A6-2679	1	
	c.53C>A	p.T18N	Hepatocellular carcinoma	CHG-15-07714 (ICGC - China)	1	
	c.62C>T	p.T21M	Germ cell tumor	AYA07 (Cha et al., *BMC Cancer*, 2016)	1	
	c.100C>A	p.P34T	Hepatocellular carcinoma	CHG-14-05691 (ICGC-China)	1	
	c.122G>A	p.R41K	Urothelial carcinoma	TCGA-DK-AA76	1	
	c.128G>T	p.S43I	Lung adenocarcinoma	TCGA-86-8585	1	
	c.137C>A	p.T46K	Hepatocellular carcinoma	CHG-14-25457 (ICGC - China)	1	
	c.169C>A	p.Q57K	Hepatocellular carcinoma	CHG-13-09220 (ICGC - China)	1	
	c.173A>T	p.K58M	Colorectal adenocarcinoma	Mx42 (Sjöblom et al., *Science*, 2006)	1	No
	c.174G>T	p.K58N	Colorectal adenocarcinoma metastasis	SNU175 cell line (CCLE - SNU175_LARGE_INTESTINE)	1	
	c.190G>A	p.D64N	Lung adenocarcinoma	SC_LUNG_09 (ICGC - South Korea)	1	
	c.197G>A	p.S66N	Uterine corpus endometrial carcinoma	TCGA-BS-A0UF	1	No
	c.201G>T	p.E67D	Colorectal adenocarcinoma	T593 (Giannakis et al., *Cell Rep*, 2016)	1	
	c.204A>G	p.I68M	Papillary thyroid carcinoma	WZ037T (ICGC - China)	1	
	c.211G>C	p.E71Q	Esophageal carcinoma	TCGA-VR-AA7B	1	
	c.230T>G	p.L77R	Acute lymphoblastic B cell leukemia	SJBALL020877_D1 (Roberts et al., *N Engl J Med*, 2014)	1	
	**c.245C>T**	**p.P82L**	**Cutaneous melanoma**	**TCGA-EE-A2GB; MELA-0201 (ICGC - Australia)**	**2**	**Partial**
	c.262G>A	p.D88N	Leiomyosarcoma	LMS23 (ICGC - France)	1	
	c.265C>A	p.L89I	Esophageal carcinoma	TCGA-JY-A6FE	1	No
	c.268C>T	p.L90F	Hepatocellular carcinoma	TCGA-ZS-A9CD	1	
	c.272C>T	p.A91V	Hepatocellular carcinoma	RK261 (ICGC - Japan)	1	No
	c.284C>A	p.A95D	Hepatocellular carcinoma	CHG-12-09086 (ICGC - China)	1	
	c.286C>G	p.L96V	Breast invasive carcinoma	TCGA-A2-A0SY	1	No
	c.294G>T	p.W98C	Hepatocellular carcinoma	SNU387 cell line (CCLE - SNU387_LIVER)	1	
	c.315G>T	p.K105N	Uterine corpus endometrial carcinoma	TCGA-BS-A0UF	1	No
	c.317G>T	p.S106I	Uterine corpus endometrial carcinoma	TCGA-D1-A17Q	1	No
	c.322C>T	p.P108S	Cutaneous melanoma	CP66-MEL cell line (CCLE - CP66MEL_SKIN)	1	
	c.331G>C	p.D111H	Prostate adenocarcinoma	0218_CRUK_PC_0218 (ICGC - UK)	1	
	c.334C>T	p.L112F	Hepatocellular carcinoma	HCC 66 (An et al., *Pathol Res Pract*, 2011)	1	No
	c.340C>T	p.H114Y	Papillary thyroid carcinoma	WZ041 (ICGC - China)	1	
	c.346_347CC>TT	p.P116L	Squamous cell carcinoma	CSCC-55 (Pickering et al., *Clin Cancer Res*, 2014)	1	No
	c.350C>A	p.S117Y	Hepatocellular carcinoma	CHG-14-18899T (ICGC - China)	1	
	c.359C>A	p.A120E	Hepatocellular carcinoma	CHG-14-15016 (ICGC - China)	1	
	c.359C>T	p.A120V	Prostate adenocarcinoma	TCGA-XK-AAIW	1	No
	c.370C>T	p.H124Y	Lung adenocarcinoma	TCGA-55-8510	1	No
	c.397G>A	p.D133N	Cervical squamous cell carcinoma	TCGA-Q1-A730	1	No
	c.413A>C	p.K138T	Uterine corpus endometrial carcinoma	TCGA-E6-A1LX	1	
	c.421G>A	p.V141I	Esophageal squamous cell carcinoma	KYSE270 cell line (CCLE - KYSE270_OESOPHAGUS)	1	
	c.422T>C	p.V141A	Uterine corpus endometrial carcinoma	TCGA-E6-A1LX	1	
	c.430G>C	p.E144Q	Lung adenocarcinoma	TCGA-55-A4DF	1	
	c.449A>G	p.H150R	Colorectal adenocarcinoma	HCT15 cell line (Mouradov et al., *Cancer Res*, 2014)	1	
	c.453G>T	p.K151N	Lung squamous cell carcinoma	TCGA-33-A5GW	1	
	c.454C>A	p.Q152K	Hepatocellular carcinoma	CHG-13-19092 (ICGC - China)	1	

c.457C>A	p.L153I	Hepatocellular carcinoma	CHG-13-14474 (ICGC - China)	1	
c.462G>T	p.W154C	Papillary thyroid carcinoma	PTC_14 (ICGC - Saudi Arabia)	1	No
c.463A>G	p.M155V	Lower grade glioma	TCGA-DU-7304	1	No
c.466G>A	p.G156R	Urothelial carcinoma	TCGA-K4-AAQO	1	
c.474A>C	p.Q158H	Uterine corpus endometrial carcinoma	TCGA-EO-A3B0	1	
c.482G>T	p.R161I	Undifferentiated Pleomorphic Sarcoma; Small cell lung cancer metastasis	TCGA-SG-A6Z4; NCI-H740 cell line (CCLE - NCIH740_LUNG)	2	
c.488A>T	p.D163V	Lung squamous cell carcinoma	TCGA-22-4591	1	
c.499G>A	p.A167T	Colorectal adenocarcinoma	CL-34 cell line (CCLE - CL34_LARGE_INTESTINE)	1	
c.508C>T	p.R170W	Colorectal adenocarcinoma	T561 (Giannakis et al., *Cell Rep*, 2016)	1	
c.517A>G	p.M173V	Endometrial adenosquamous carcinoma	MFE-319 cell line (CCLE - MFE319_ENDOMETRIUM)	1	
c.526C>A	p.P176T	Hepatocellular carcinoma	CHG-13-29153 (ICGC - China)	1	
c.529G>C	p.A177P	Squamous cell carcinoma metastasis; Burkitt's lymphoma	BICR 18 cell line (CCLE - BICR18_UPPER_AERODIGESTIVE_TRACT); DAUDI cell line (CCLE - DAUDI_HAEMATOPOIETIC_AND_LYMPHOID_TISSUE)	2	
c.541G>A	p.G181R	Gastric adenocarcinoma	GCYC_410 (ICGC - Japan)	1	
c.547C>T	p.R183C	Cutaneous melanoma	TCGA-EE-A2GB; MELA-0167 (ICGC- Australia)	2	No
c.548G>A	p.R183H	T-cell acute lymphoblastic leukemia	MOLT-13 cell line (CCLE - MOLT13_HAEMATOPOIETIC_AND_LYMPHOID_TISSUE)	1	
**c.556C>T**	**p.P186S**	**Cutaneous melanoma**	**TCGA-GN-A269**	**1**	**Partial**
c.556C>A	p.P186T	Hepatocellular carcinoma	CHG-13-30014 (ICGC - China)	1	
c.577A>T	p.T193S	Breast invasive carcinoma	TCGA-E9-A3HO	1	No
c.578C>T	p.T193M	Nasopharyngeal carcinoma	HKNPC-042-Tumor-SM-64MG9 (Li et al., *Nat Commun*, 2017)	1	
c.580A>T	p.T194S	Breast invasive carcinoma	TCGA-E9-A3HO	1	No
c.604C>A	p.L202M	Hepatocellular carcinoma	CHG-15-10295 (ICGC - China)	1	
c.610C>A	p.R204S	Hepatocellular carcinoma	CHG-14-28278 (ICGC - China)	1	
c.610C>T	p.R204C	Gastric adenocarcinoma	IM95 cell line (CCLE - IM95_STOMACH)	1	
c.611G>A	p.R204H	Gastric adenocarcinoma; endometrial adenocarcinoma; colorectal adenocarcinoma	pfg100T (Wang et al., *Nature Genetics*, 2014); HEC-1 cell line (CCLE - HEC1_ENDOMETRIUM); SW48 cell line (Mouradov et al., *Cancer Res*, 2014)	3	No
c.622G>A	p.A208T	Uterine corpus endometrial carcinoma	TCGA-BG-A0M4	1	No
c.628G>C	p.G210R	Renal clear cell carcinoma	TCGA-B8-A54I	1	
c.629G>T	p.G210V	Renal clear cell carcinoma	TCGA-B8-A54I	1	
c.631C>A	p.Q211K	Hepatocellular carcinoma	CHG-14-18899T (ICGC - China)	1	
c.639C>A	p.H213Q	Hepatocellular carcinoma	CHG-14-03368; CHG-15-10295 (ICGC - China)	2	
c.641C>T	p.T214I	Colorectal adenocarcinoma	SNU-1040 cell line (CCLE - SNU1040_LARGE_INTESTINE)	1	
c.650A>T	p.D217V	Esophageal carcinoma	TCGA-L5-A4OM	1	
c.658A>C	p.K220Q	Thyroid medullary carcinoma	TT cell line (CCLE - TT_THYROID)	1	
c.671C>T	p.P224L	Cutaneous melanoma	MZ-MEL-7 cell line (CCLE - MZ7MEL_SKIN)	1	
c.674C>T	p.S225F	Invasive urothelial bladder carcinoma	TCGA-DK-A1AC	1	No
c.682G>C	p.D228H	Salivary duct carcinoma	SDC16 (Dalin et al., *Clin Cancer Res*, 2016)	1	
c.688G>A	p.E230K	Colorectal adenocarcinoma	TCGA-AA-A017	1	No
c.695G>A	p.G232E	Prostate neuroendocrine carcinoma metastasis	MO_1462 (Robinson et al., Nature, 2017)	1	
c.697G>A	p.E233K	Colorectal adenocarcinoma; Uterine corpus endometriod carcinoma	8804_CLM; 4989_CLM; 4989_PT; 2557_PT (Lim et al., *Oncotarget*, 2015); TCGA-AJ-A2QO	5	
c.698A>G	p.E233G	Uterine corpus endometrial carcinoma	TCGA-E6-A1LX	1	
c.705G>T	p.K235N	Colorectal adenocarcinoma	HCC_2998 cell line (Mouradov et al., *Cancer Res*, 2014)	1	No
c.712G>C	p.V238L	Cervical squamous cell carcinoma	TCGA-VS-A9UP	1	
**c.721C>A**	**p.H241N**	**Uterine carcinosarcoma**	**MM04T (Jones et al., *Nat Commun.*, 2014)**	**1**	**Partial**
**c.721C>T**	**p.H241Y**	**Gastric adenocarcinoma**	**STC 253 (An et al., *Pathol Res Pract*, 2011)**	**1**	**Complete**
**c.724G>A**	**p.G242R**	**Pancreatic ductal adenocarcinoma**	**8052570 (ICGC - Australia)**	**1**	**Complete**
c.736A>G	p.M246V	Small cell lung carcinoma metastasis	NCI-H1048 cell line (CCLE - NCIH1048_LUNG)	1	
c.770A>G	p.H257R	Hepatocellular carcinoma	TCGA-MI-A75E	1	No
**c.775A>C**	**p.S259R**	**Hepatocellular carcinoma**	**TCGA-MI-A75E**	**1**	**Complete**
**c.782C>T**	**p.P261L**	**Prostate adenocarcioma metastasis; Gastric adenocarcinoma**	**03-130L (Kumar et al., *Nat Med*, 2016); TCGA-HU-8602**	**2**	**Partial**
c.787A>G	p.N263D	Colorectal adenocarcinoma	T3110 (Giannakis et al., *Cell Rep*, 2016)	1	
c.793C>A	p.L265I	Colorectal adenocarcinoma	TCGA-AA-3977	1	
c.793C>T	p.L265F	Breast invasive carcinoma	TCGA-AC-A23H	1	
c.796C>T	p.H266Y	Cutaneous melanoma	TCGA-YG-AA3O	1	
c.797A>G	p.H266R	Colorectal adenocarcinoma	T1762 (Giannakis et al., *Cell Rep*, 2016)	1	
c.803G>T	p.S268I	Germinal center B-cell derived lymphoma; Burkitt's lymphoma metastasis	4196446 (ICGC - Germany); DG-75 cell line (CCLE - DG75_HAEMATOPOIETIC_AND_LYMPHOID_TISSUE)	2	
c.810C>G	p.I270M	Prostate adenocarcinoma	CPCG0373 (ICGC - Canada)	1	
c.811C>T	p.P271S	Desmoplastic melanoma	37M (Shain et al., *Nat Genet*, 2015)	1	
c.826T>G	p.*276G	Ovarian serous adenocarcinoma metastasis	SNU-119 cell line (CCLE - SNU119_OVARY)	1	
